# Vitamin K2 promotes PI3K/AKT/HIF-1α-mediated glycolysis that leads to AMPK-dependent autophagic cell death in bladder cancer cells

**DOI:** 10.1038/s41598-020-64880-x

**Published:** 2020-05-07

**Authors:** Fengsen Duan, Chunlei Mei, Luhao Yang, Junyan Zheng, Huiai Lu, Yanzhi Xia, Stacy Hsu, Huageng Liang, Ling Hong

**Affiliations:** 1 Department of Biology, College of Life Science and Technology, Huazhong University of Science and Technology, 430074 Wuhan, China; 2 Tongji Medical College, Huazhong University of Science and Technology, 430030 Wuhan, China; 3 Department of Urology, Union Hospital, Tongji Medical College, Huazhong University of Science and Technology, 430022 Wuhan, China; 4 Global Public Health (Biology), Pre-Medicine Track, New York University College of Arts & Science 2021, 10003 New York, USA

**Keywords:** Cancer metabolism, Apoptosis, Autophagy

## Abstract

Vitamin K2 has been shown to exert remarkable anticancer activity. However, the detailed mechanism remains unclear. Here, our study was the first to show that Vitamin K2 significantly promoted the glycolysis in bladder cancer cells by upregulating glucose consumption and lactate production, whereas inhibited TCA cycle by reducing the amounts of Acetyl-CoA. Moreover, suppression of PI3K/AKT and HIF-1α attenuated Vitamin K2-increased glucose consumption and lactate generation, indicating that Vitamin K2 promotes PI3K/AKT and HIF-1α-mediated glycolysis in bladder cancer cells. Importantly, upon glucose limitation, Vitamin K2-upregulated glycolysis markedly induced metabolic stress, along with AMPK activation and mTORC1 pathway suppression, which subsequently triggered AMPK-dependent autophagic cell death. Intriguingly, glucose supplementation profoundly abrogated AMPK activation and rescued bladder cancer cells from Vitamin K2-triggered autophagic cell death. Furthermore, both inhibition of PI3K/AKT/HIF-1α and attenuation of glycolysis significantly blocked Vitamin K2-induced AMPK activation and subsequently prevented autophagic cell death. Collectively, these findings reveal that Vitamin K2 could induce metabolic stress and trigger AMPK-dependent autophagic cell death in bladder cancer cells by PI3K/AKT/HIF-1α-mediated glycolysis promotion.

## Introduction

Cancer cells, including bladder carcinoma cells, display the altered metabolism, compared to normal cells^1^. One of the most metabolic shifts in cancer cells is the aberrant glucose metabolism. Unlike the normal cells, most cancer cells exhibit the remarkably increased glucose uptake and glycolysis rate to meet their rapid proliferation and metastasis^2^. Moreover, numerous studies indicate that the glycolysis is usually uncoupled from the tricarboxylic acid (TCA) cycle and oxidative phosphorylation (OXPHOS) in cancer cells. Therefore, pyruvate, the end product of glycolysis, is mainly diverted to lactate production, with the reduction of mitochondrial TCA cycle and OXPHOS^3^. This metabolic shift is well-known as the Warburg effect.

However, in tumor microenvironment, the nutrients including glucose are limited. Excessively increasing glycolysis will inevitably result in intracellular metabolic stress and trigger cancer cell death due to energy depletion^4^. Therefore, in nutrient-deficient tumor microenvironment, the method of promoting glycolysis to induce metabolic stress and activate cell death appears to be a novel strategy for cancer treatment.

Phosphatidylinositide-3-kinase (PI3K) and AKT are usually hyper-activated in cancer cells. There are accumulating evidences indicating that activation of PI3K and AKT plays a pivotal role in the regulation of aerobic glycolysis in cancer cells^5–7^. The activated PI3K/AKT can directly promote the shift to the aerobic glycolysis, rendering cancer cells more reliance on glucose consumption for lactate generation^8,9^. Although activation of PI3K and AKT could directly stimulate the aerobic glycolysis by positive regulation of some glycolytic proteins or enzymes, such as glucose transporters, Hexokinase II (HK2) and Lactate Dehydrogenase (LDH), it also renders cancer cells more susceptible to death upon glucose starvation^10,11^. Hypoxia-inducible factor-1α (HIF-1α) has recently been found to participate in promoting the glycolytic metabolism in cancer cells. Under hypoxic conditions, HIF-1α promotes the shift to glycolysis by upregulating pyruvate dehydrogenase kinase 1 (PDHK1) which in turn inactivates pyruvate dehydrogenase (PDH), resulting in the inhibition of TCA cycle and mitochondrial OXPHOS, and leading to PDHK1-dependent glucose metabolism reprogramming^12–14^.

The AMP-activated protein kinase (AMPK) is a highly conserved metabolic regulator, which maintains energy homeostasis during metabolic stress. Upon energy shortage, AMPK is rapidly activated and restores the energy balance by inhibiting the anabolic processes and simultaneously promoting the catabolic processes^15–17^. In addition, glucose deprivation also triggers AMPK activation by sensing the absence of fructose-1, 6-bisphosphate (FBP)^18^. Autophagy is another catabolic pathway not only for maintenance of intracellular homeostasis, but also for adaptive responses to metabolic stress^19,20^. There are increasing evidences suggesting that activated AMPK can stimulate autophagy not only by direct activation of ULK1 (unc-51-like autophagy-activating kinase 1), but also by suppression of mTORC1^21,22^. Upon energy shortage, autophagy initiated by AMPK/ULK1-mTORC1 mode usually determines two opposite cell fates, survival and death. In some circumstances, appropriate autophagy is sufficient to provide ATP to meet intracellular energy demands for cell survival. Conversely, excessive autophagy could promote apoptosis and result in autophagic cell death^23–26^.

Vitamin K2 (menaquinone) is a natural product from a vast variety of bacteria and originally isolated from putrefied fishmeal^27^. Recently, Vitamin K2 has been shown to exert remarkable anticancer activity in various cancer cell lines^28–31^. However, the detailed anticancer mechanism of Vitamin K2 remains elusive. In this study, we aimed to establish the metabolic signal axis: PI3K/AKT/HIF-1α dependent glycolysis promotion, metabolic stress, AMPK activation and mTORC1 suppression, which induces autophagic cell death in bladder cancer cells and reveals the exact anticancer mechanism of Vitamin K2.

## Results

### Vitamin K2 promotes glycolysis and inhibits TCA cycle in bladder cancer cells

**Figure 1:**
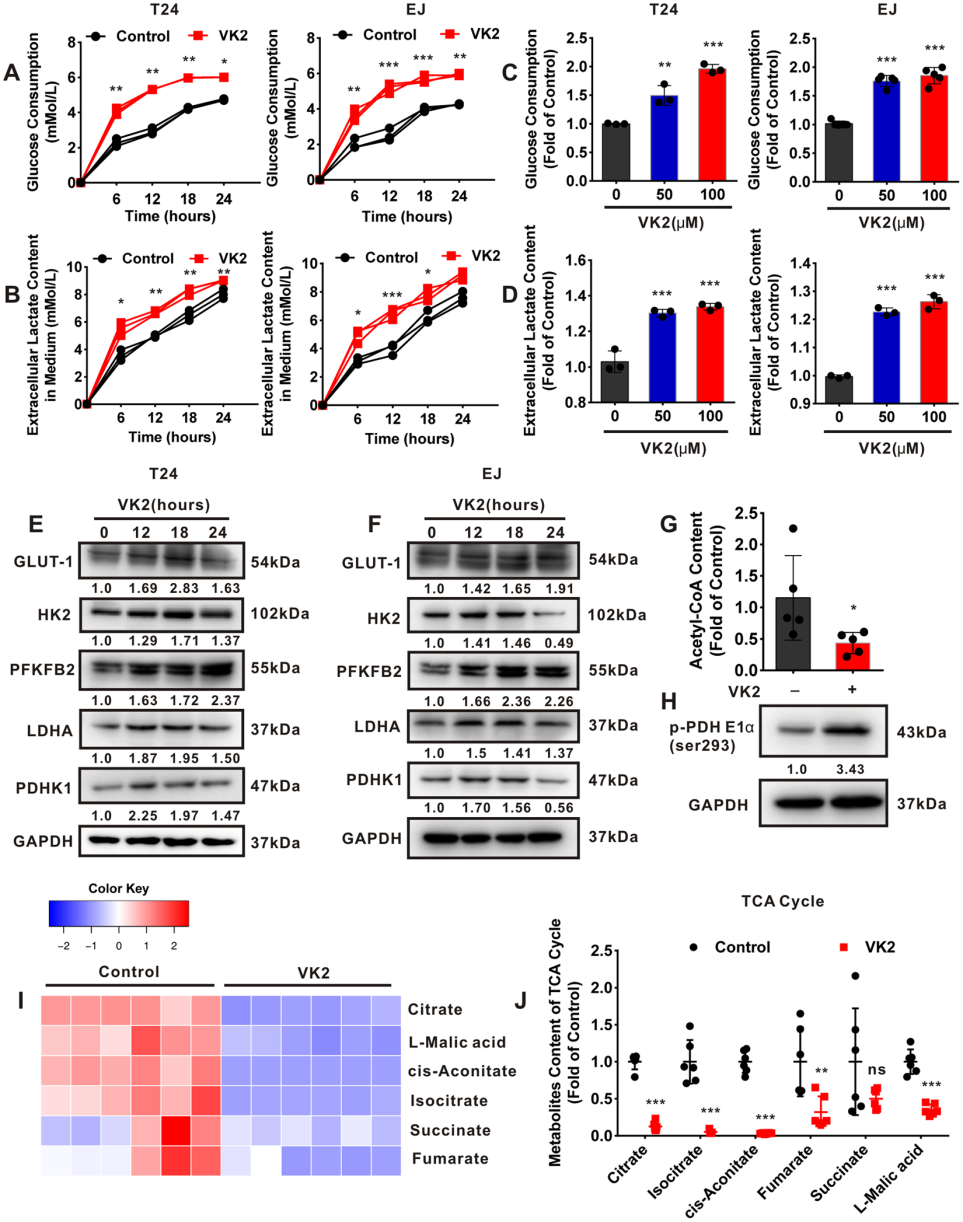
Vitamin K2 promotes glycolysis and inhibits TCA cycle in bladder cancer cells. (**A–D**) Vitamin K2 time or dose-dependently affected the glucose consumption and lactate production in bladder cancer T24 and EJ cells. (**E**) and (**F**) Western blot showed the effect of 50 μM Vitamin K2 on the expression of glycolytic proteins or enzymes in bladder cancer T24 and EJ cells. (**G**) T24 cells were exposed to 50 μM Vitamin K2 for 12 hours and the quantitative analysis of Acetyl-CoA amount was measured by LC-MS. (**H**) Western blot showed the effect of 50 μM Vitamin K2 on the phosphorylation of PDH in T24 cells. (**I**) and (**J**) T24 cells were exposed to 50 μM Vitamin K2 for 12 hours and the quantitative analysis of the metabolites content in TCA cycle was measured by LC-MS. Data are presented as the mean ± SD of at least three independent experiments. *P < 0.05, **P < 0.01 and ***P < 0.001.

To evaluate the effect of Vitamin K2 on the glucose metabolism in bladder cancer cells, the glycolytic process was examined. In the present study, Vitamin K2 remarkably promoted the glucose consumption and lactate production in bladder cancer T24, EJ and J82 cells in a time or dose-dependent manner (Fig. 1A–D and Supplementary Fig. 1). Moreover, Western blot analysis showed that Vitamin K2 stepwise elevated the expression of some glycolytic proteins or enzymes, such as GLUT-1, Hexokinase II (HK2), PFKFB2, LDHA and PDHK1, in bladder cancer T24 (Fig. 1E) and EJ cells (Fig. 1F). These results indicate that Vitamin K2 promotes the glycolysis in bladder cancer cells. We next assessed the effect of Vitamin K2 on the tricarboxylic acid (TCA) cycle in bladder cancer cells. Comparing to the control group, Vitamin K2 remarkably decreased the amounts of Acetyl coenzyme A (Acetyl-CoA) in T24 cells (Fig. 1G). Furthermore, pyruvate dehydrogenase (PDH) was notably phosphorylated in Vitamin K2-treated T24 cells, suggesting that Vitamin K2 inactivates PDH (Fig. 1H). To determine the inhibitory effect of Vitamin K2 on TCA cycle in bladder cancer cells, the contents of key metabolites were detected. As shown in Fig. 1I,J, Vitamin K2 markedly reduced the amounts of metabolites in TCA cycle, compared with the control group, implying that Vitamin K2 suppresses the TCA cycle in bladder cancer cells. Taken together, our results reveal that Vitamin K2 renders bladder cancer cells more dependence on glycolysis than TCA cycle.

### Activation of PI3K/AKT and HIF-1α contributes to Vitamin K2-upregulated glycolysis in bladder cancer cells

**Figure 2:**
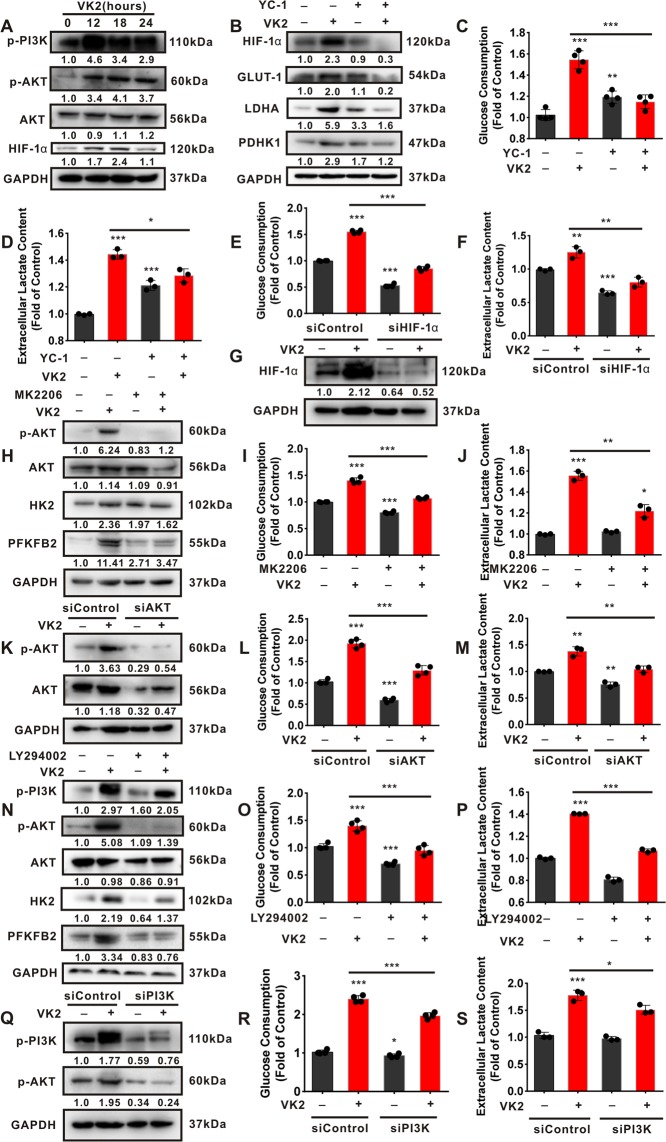
Vitamin K2 upregulates the glycolysis in bladder cancer cells by stimulating PI3K/AKT and HIF-1α signal pathways. (**A**) Western blot demonstrated that 50 μM Vitamin K2 affected the activation of PI3K/AKT and the expression of HIF-1α in T24 cells. (**B**) Western blot showed the effect of 100 μM YC-1 on the expression of HIF-1α, GLUT-1, LDHA and PDHK1 in T24 cells treated with or without 50 μM Vitamin K2. (**C**), (**D**), (**I**), (**J**), (**O**) and (**P**) The effect of YC-1, 4 μM MK2206 and 50 μM LY294002 on the glucose consumption and lactate production in T24 cells treated with or without Vitamin K2. (**G**) Western blot showed the effect of HIF-1α siRNA on the expression of HIF-1α in T24 cells treated with or without Vitamin K2. (**E**), (**F**), (**L**), (**M**), (**R**) and (**S**) The effect of HIF-1α siRNA, AKT siRNA and PI3K siRNA on the glucose consumption and lactate generation in Vitamin K2-treated T24 cells. (**H**) and (**N**) Western blot demonstrated the effect of MK2206 and LY294002 on the expression of AKT, HK2 and PFKFB2 in Vitamin K2-treated T24 cells. (**K**) and (**Q**) Western blot showed the effect of AKT siRNA and PI3K siRNA on the expression of AKT in T24 cells treated with or without Vitamin K2. Data are presented as the mean ± SD of three or four independent experiments. *P < 0.05, **P < 0.01 and ***P < 0.001.

We next investigate whether activation of PI3K/AKT and HIF-1α is implicated in Vitamin K2-upregulated glycolysis in bladder cancer cells. As shown in Fig. 2A, Vitamin K2 significantly induced the phosphorylation of PI3K/AKT, and increased HIF-1α expression in bladder cancer T24 cells in a time-dependent manner. Moreover, both YC-1 (a pharmacological inhibitor of HIF-1α) and HIF-1α siRNA significantly attenuated Vitamin K2-elevated glucose consumption and lactate production in T24 cells (Fig. 2B–G). To validate the involvement of PI3K/AKT activation in Vitamin K2-induced glycolysis promotion, MK2206 (a specific inhibitor of AKT) and LY294002 (a typical PI3K inhibitor) were respectively utilized. Our data showed that both MK2206 and LY294002 markedly alleviated Vitamin K2-increased glucose consumption and lactate production in T24 (Fig. 2H–J,N–P) and EJ cells (Supplementary Fig. 2). Additionally, Vitamin K2-upregulated glucose consumption and lactate production were significantly inhibited by respective treatment with AKT siRNA and PI3K siRNA (Fig. 2K–M and Fig. 2Q–S). Collectively, these results indicate that Vitamin K2 promotes PI3K/AKT and HIF-1α-mediated glycolysis in bladder cancer cells.

### Vitamin K2 induces metabolic stress in bladder cancer cells

**Figure 3:**
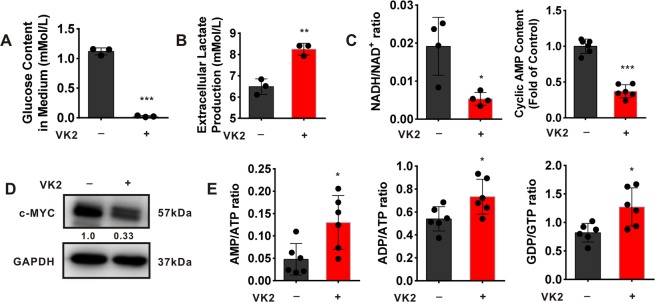
Vitamin K2 induces metabolic stress in bladder cancer cells. (**A,B**) The changes of glucose content and lactate production in T24 cell culture medium after treatment with or without 50 μM Vitamin K2 for 18 hours. (**C,E**) The alteration of intracellular energy status in T24 cells after exposed to 50 μM Vitamin K2 for 18 hours. (**D**) Western blot demonstrated the changes of c-MYC expression in T24 cells after exposed to 50 μM Vitamin K2 for 18 hours. Data are presented as the mean ± SD of at least three independent experiments. *P < 0.05, **P < 0.01 and ***P < 0.001.

We next investigate whether Vitamin K2-upregulated glycolysis results in metabolic stress, upon glucose limitation. Our results showed that glucose in T24 cell culture medium was almost depleted, and lactate generation attained the maximum after exposed to Vitamin K2 for 18 hours (Fig. 3A,B). To determine the metabolic stress resulted from Vitamin K2-elevated glycolysis in bladder cancer cells, we assessed the NADH:NAD^+^ ratio, cyclic AMP content and c-MYC expression whose alteration are susceptible to glucose starvation^32,33^. As shown in Fig. 3C, both the ratio of NADH:NAD^+^ and content of cyclic AMP were dramatically decreased in T24 cells after exposed to Vitamin K2 for 18 hours. Moreover, c-MYC protein level was also significantly reduced in T24 cells following treatment with Vitamin K2 for 18 hours (Fig. 3D). In addition, As shown in Fig. 3E, the ratios of AMP:ATP, ADP:ATP and GDP:GTP were markedly increased in T24 cells after exposed to Vitamin K2 for 18 hours. These results suggest that Vitamin K2 is able to induce metabolic stress, including glucose starvation and energy shortage, in bladder cancer cells, upon glucose limitation.

### Vitamin K2 triggers autophagic cell death in bladder cancer cells

**Figure 4:**
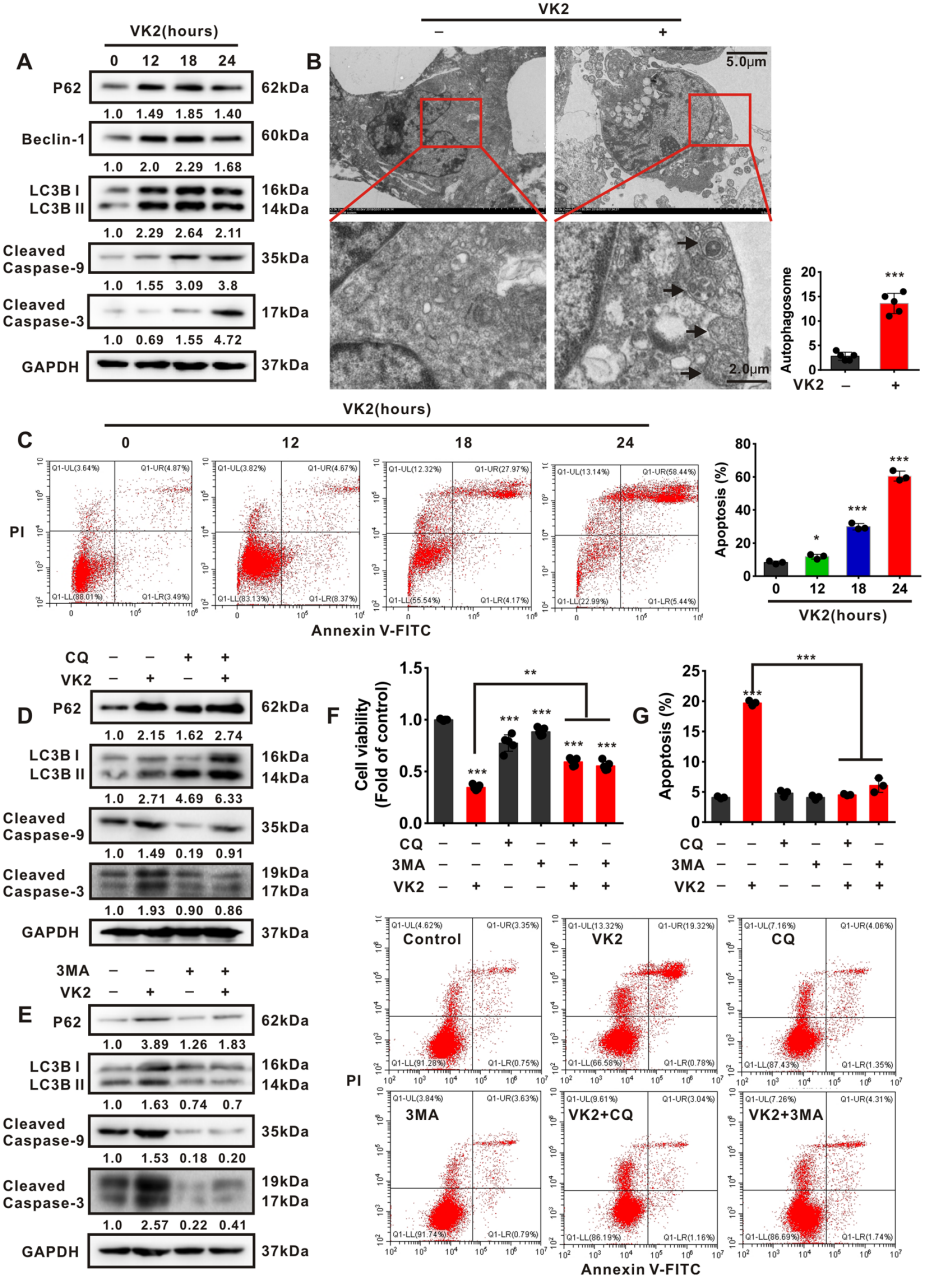
Vitamin K2 triggers autophagic cell death in bladder cancer cells. (**A**) Western blot demonstrated the effect of 50 μM Vitamin K2 on the expression of autophagic and apoptotic proteins in T24 cells. (**B**) Electronic microscope showed the formation of autophagosomes in T24 cells after treatment with or without 50 μM Vitamin K2 for 18 hours. Arrow pointed to autophagosomes. Scale bar: upper: 5.0μm, lower: 2.0μm. (**C**) Flow cytometry demonstrated the apoptotic effect of Vitamin K2 on T24 cells. (**D,E**) Western blot demonstrated the effect of 50 μM CQ and 6 mM 3MA on the expression of autophagic and apoptotic proteins in T24 cells treated with or without 50 μM Vitamin K2. (**F**) MTS assays demonstrated the effect of CQ and 3MA on the cell viability in Vitamin K2-treated T24 cells. (**G**) Flow cytometry showed the effect of CQ and 3MA on apoptosis in T24 cells treated with or without Vitamin K2. Data are presented as the mean ± SD of at least three independent experiments. *P < 0.05, **P < 0.01 and ***P < 0.001.

To investigate whether Vitamin K2-induced metabolic stress subsequently triggers autophagic cell death, the autophagic and apoptotic effect of Vitamin K2 on bladder cancer cells were respectively evaluated. Our data showed that Vitamin K2 significantly upregulated the expression of P62, Beclin-1, LC3B II, and induced the cleavage of Caspase-9 and Caspase-3 in T24 (Fig. 4A) and EJ cells (Supplementary Fig. 3B) in a time-dependent manner. Moreover, the autophagosomes and prominent punctate LC3 were observed in Vitamin K2-treated T24 cells (Fig. 4B and Supplementary Fig. 3A). In addition, flow cytometry showed that the apoptotic rate was remarkably increased in T24 (Fig. 4C) and EJ cells (Supplementary Fig. 3C) after extended exposure to Vitamin K2. To determine the autophagic cell death, we evaluated the effect of autophagy on apoptosis in Vitamin K2-treated bladder cancer cells, and chloroquine (CQ) and 3-Methyladenine (3-MA), two typical autophagic inhibitors, were respectively employed. As shown in Fig. 4D–G, both CQ and 3-MA markedly suppressed autophagy, and blocked apoptosis in Vitamin K2-treated T24 cells. Furthermore, Rapamycin, a specific autophagic agonist, significantly activated autophagy, and enhanced Vitamin K2-triggered apoptosis in T24 cells (Supplementary Fig. 3D,E). These results indicate that Vitamin K2-triggered autophagy promotes the subsequent apoptosis, and ultimately results in autophagic cell death in bladder cancer cells, upon metabolic stress.

### Vitamin K2 triggers AMPK-dependent autophagic cell death in bladder cancer cells

**Figure 5:**
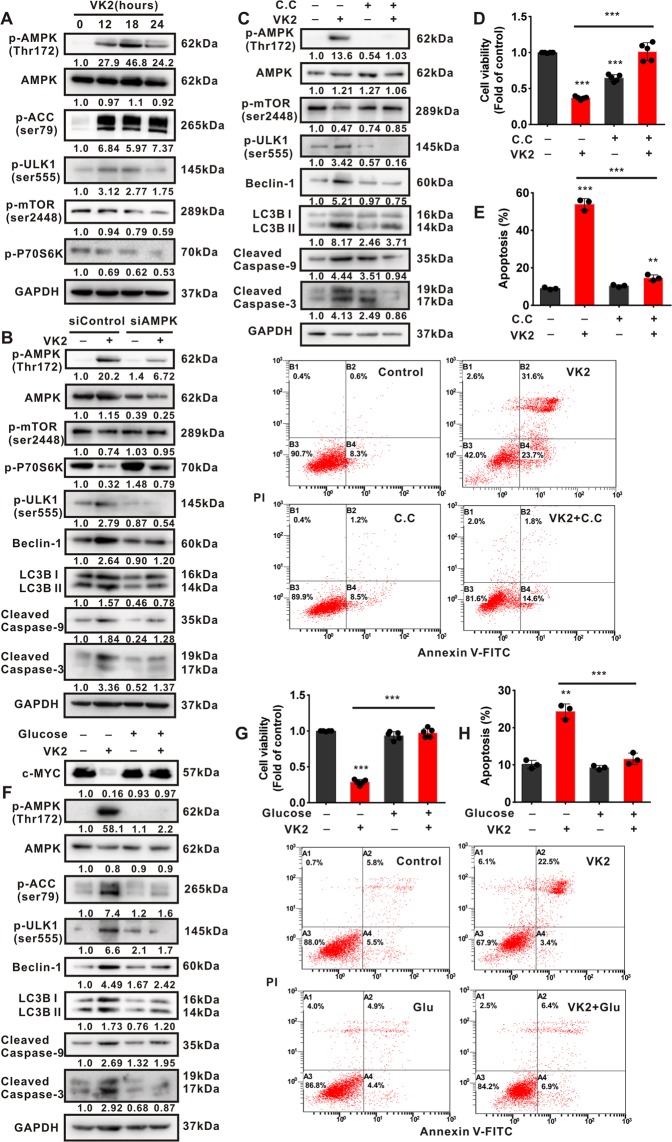
Vitamin K2 induces AMPK-dependent autophagic cell death in bladder cancer cells. (**A**) Western blot showed the effect of 50 μM Vitamin K2 on activation of AMPK and mTORC1 pathway in T24 cells. (**B**) Western blot showed the effect of AMPK siRNA on the activation of AMPK, mTORC1, autophagy and apoptosis in Vitamin K2-treated T24 cells. (**C**) Western blot showed the effect of 40 μM Compound C (C.C) on the activation of AMPK, mTORC1, autophagy and apoptosis in Vitamin K2-treated EJ cells. (**D,G**) MTS assays demonstrated the effect of Compound C and 10 mM glucose supplementation on the cell viability in Vitamin K2-treated EJ or T24 cells. (**E,H**) Flow cytometry demonstrated the effect of Compound C and glucose supplementation on apoptosis in Vitamin K2-treated EJ or T24 cells. (**F**) Western blot showed the effect of glucose supplementation on c-MYC expression and activation of AMPK, autophagy and apoptosis in T24 cells treated with or without Vitamin K2. Data are presented as the mean ± SD of at least three independent experiments. *P < 0.05, **P < 0.01 and ***P < 0.001.

Given that AMPK maintains energy homeostasis in response to energy shortage or metabolic stress, we next investigated whether AMPK was activated due to Vitamin K2-induced metabolic stress in bladder cancer cells. Our results showed that Vitamin K2 markedly increased the phosphorylation of AMPK, ACC1 (Acetyl-CoA-Carboxylase 1) and ULK1 (unc-51-like autophagy-activating kinase 1), whereas reduced the phosphorylation of mTORC1 (mammalian target of rapamycin complex 1) and P70S6K (one of the downstream of mTORC1), in T24 (Fig. 5A) and EJ cells (Supplementary Fig. 4A). These results suggest that Vitamin K2 could activate AMPK pathway and conversely suppress mTORC1 pathway in bladder cancer cells in response to metabolic stress. To investigate whether Vitamin K2-triggered autophagic cell death are AMPK dependent, the inhibition of AMPK was performed. Our data showed that both AMPK siRNA and Compound C (C.C, a pharmacological inhibitor of AMPK) were able to abrogate the activation of AMPK pathway and simultaneously restore mTORC1 pathway, which subsequently suppressed autophagic cell death in Vitamin K2-treated bladder cancer cells (Fig. 5B–E). These results reveal that Vitamin K2 could trigger AMPK-dependent autophagic cell death in bladder cancer cells, upon metabolic stress. To determine whether Vitamin K2-induced metabolic stress is responsible for AMPK-dependent autophagic cell death in bladder cancer cells, glucose supplementation was performed to prevent metabolic stress. Intriguingly, glucose supplementation completely restored c-MYC expression, abrogated AMPK pathway activation and markedly rescued bladder cancer cells from autophagic cell death (Fig. 5F–H and Supplementary Fig. 4B,C). Taken together, these results indicate that Vitamin K2 is able to trigger AMPK-dependent autophagic cell death in bladder cancer cells, upon metabolic stress.

### Activation of PI3K/AKT and HIF-1α is crucial for Vitamin K2-triggered AMPK-dependent autophagic cell death in bladder cancer cells

**Figure 6:**
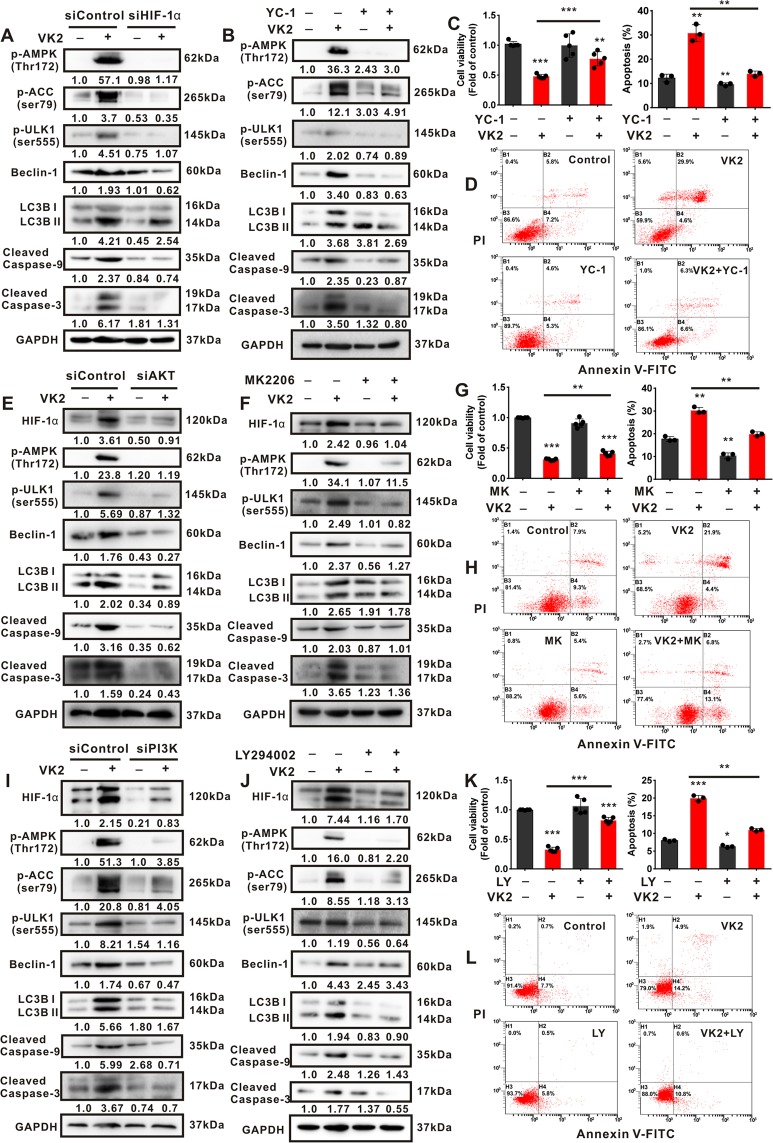
Vitamin K2 triggers AMPK-dependent autophagic cell death in T24 cells via PI3K/AKT/HIF-1α signal pathways. (**A,B**) Western blot analysis showed the effect of HIF-1α siRNA or YC-1 on the activation of AMPK pathway, autophagy and apoptosis in Vitamin K2-treated T24 cells. (**C,G,K**) MTS assays demonstrated the effect of YC-1, MK2206 and LY294002 on the reduced T24 cell viability triggered by Vitamin K2. (**D,H,L**) Flow cytometry determined the influence of YC-1, MK2206 and LY294002 on the Vitamin K2-induced apoptosis in T24 cells. (**E,F,I,J**) Western blot showed the effect of AKTsiRNA, MK2206, PI3K siRNA and LY294002 on the activation of AMPK pathways, autophagy and apoptosis in Vitamin K2-treated T24 cells. Data are presented as the mean ± SD of at least three independent experiments. *P < 0.05, **P < 0.01 and ***P < 0.001.

To investigate whether activation of PI3K/AKT and HIF-1α is involved in Vitamin K2-triggered AMPK-dependent autophagic cell death, the inhibition of PI3K/AKT and HIF-1α were respectively performed. Our data showed that both HIF-1α siRNA and YC-1 significantly blocked the activation of AMPK pathway, and inhibited the autophagic cell death in Vitamin K2-treated T24 cells (Fig. 6A–D), suggesting that Vitamin K2-increased HIF-1α expression is greatly responsible for AMPK-dependent autophagic cell death in bladder cancer cells. Moreover, both HIF-1α elevation and AMPK-dependent autophagic cell death induced by Vitamin K2 were remarkably suppressed by respective treatment with AKT siRNA (Fig. 6E), MK2206 (Fig. 6F–H), PI3K siRNA (Fig. 6I) and LY294002 (Fig. 6J–L and Supplementary Fig. 5). These results reveal that PI3K/AKT locates in the upstream of HIF-1α and contributes to Vitamin K2-triggered AMPK-dependent autophagic cell death in bladder cancer cells. Taken together, Vitamin K2 triggers AMPK-dependent autophagic cell death in bladder cancer cells via PI3K/AKT and HIF-1α signal pathways.

### Attenuation of glycolysis prevents Vitamin K2-triggered AMPK dependent autophagic cell death in bladder cancer cells

**Figure 7:**
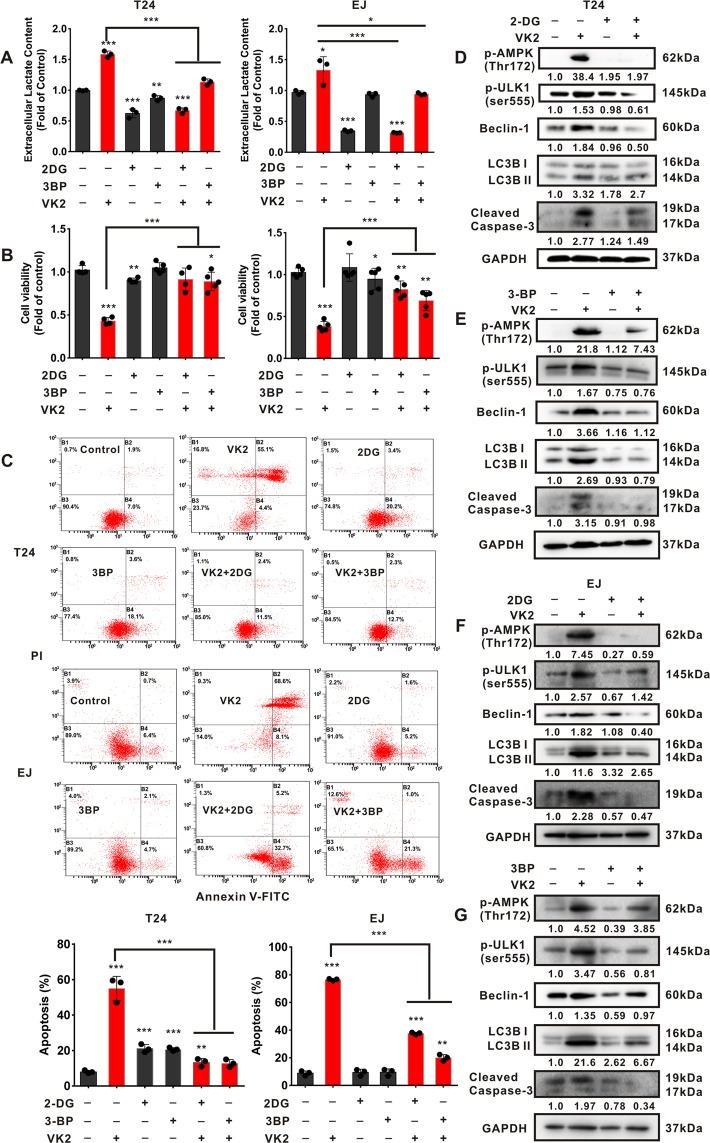
Attenuation of glycolysis blocks AMPK-dependent autophagic cell death in Vitamin K2-treated bladder cancer cells. (**A**) The effect of 5 mM 2-DG and 75 μM 3-BP on the lactate production in Vitamin K2-treated T24 or EJ cells. (**B**) The effect of 2-DG and 3-BP on the cell viability of T24 or EJ cells treated with or without 50 μM Vitamin K2. (**C**) Flow cytometry demonstrated the effect of 2-DG and 3-BP on apoptosis in T24 or EJ cells treated with or without Vitamin K2. (**D–G**) Western blot analysis showed the effect of 2-DG and 3-BP on AMPK activation, autophagy and apoptosis in Vitamin K2-treated T24 or EJ cells. Data are presented as the mean ± SD of at least three independent experiments. *P < 0.05, **P < 0.01 and ***P < 0.001.

To validate that AMPK-dependent autophagic cell death is due to Vitamin K2-induced glycolysis promotion, 2-Deoxy-D-glucose (2-DG), Sodium dichloroacetate (DCA) and 3-Bromopyruvic acid (3-BP), three typical glycolytic inhibitors, were employed. As shown in Fig. 7A,B and Supplementary Fig. 6A,B, 2-DG, 3-BP and DCA respectively attenuated Vitamin K2-upregulated glycolysis, and reversed the decreased cell viability in T24 and EJ cells. Furthermore, Vitamin K2-triggered apoptosis in T24 and EJ cells was profoundly abolished by respective treatment with 2-DG and 3-BP (Fig. 7C). Concordantly, western blot showed that Vitamin K2-induced activation of AMPK, autophagy and apoptosis were significantly blocked by treatment with 2-DG, 3-BP and DCA, respectively (Fig. 7D–G and Supplementary Fig. 6C). These results indicate that Vitamin K2-upregulated glycolysis accounts for AMPK-dependent autophagic cell death in bladder cancer cells.

### Vitamin K2 triggers AMPK-dependent autophagic cell death *in vivo*

**Figure 8:**
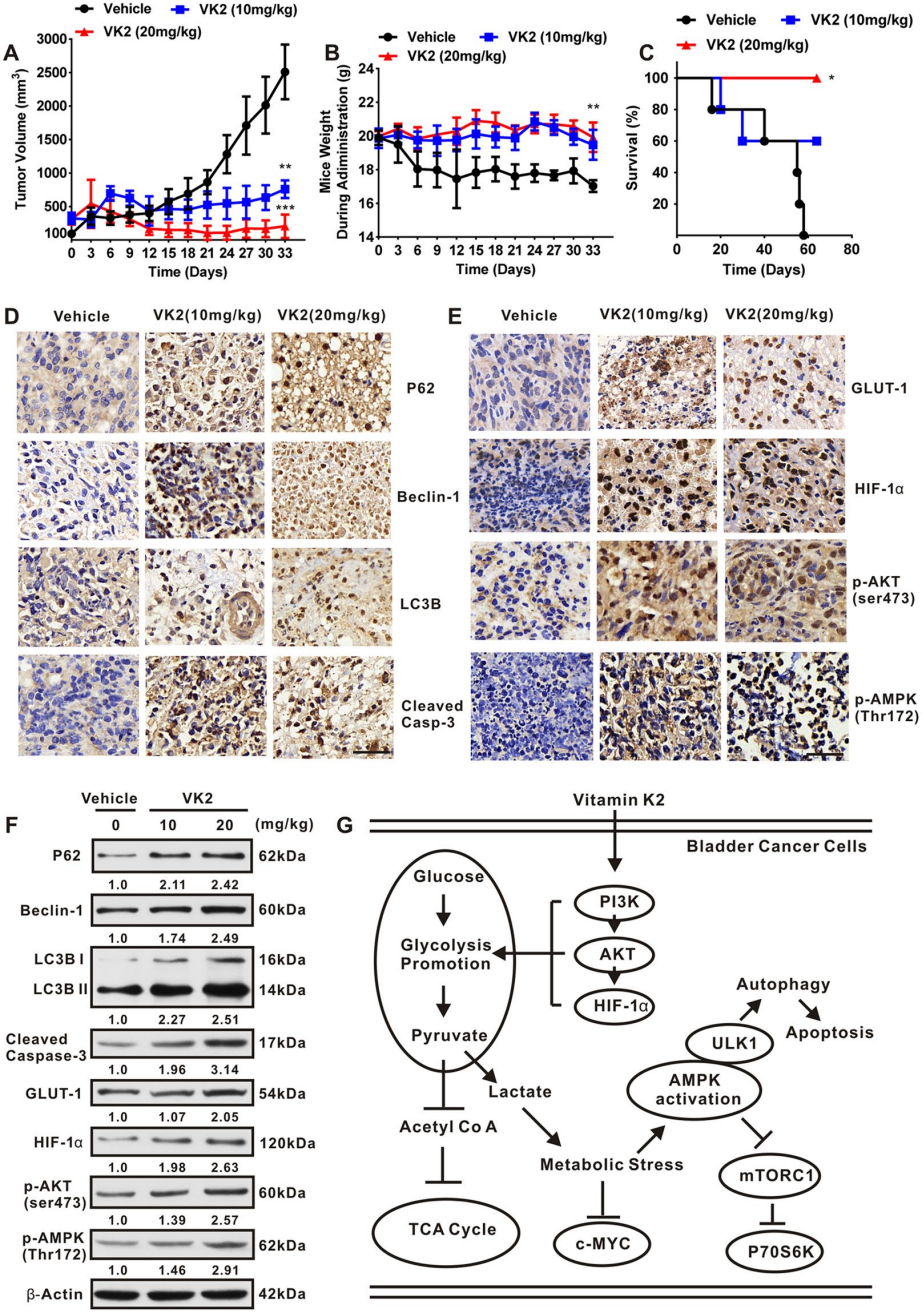
Vitamin K2 triggers AMPK-dependent autophagic cell death *in vivo*. (**A**) Tumor volume changed during administration with or without 10, 20 mg/kg Vitamin K2 every day for 33 days. (**B**) Mice weight changed during administration with or without 10, 20 mg/kg Vitamin K2. (**C**) The mice survival rates after administration with or without Vitamin K2. (**D,E**) Immunohistochemistry (IHC) showed the changes of relevant proteins expression in Vitamin K2-treated tumors, scale bar: 20 μm. (**F**) Western blot showed the effect of Vitamin K2 on the expression of relevant proteins in tumors. (**G**) The schematic diagram of metabolic signal axis involved in Vitamin K2-induced AMPK dependent autophagic cell death. Data represent the mean ± SD of three different experiments with triplicate sets in each assay. *P < 0.05, **P < 0.01 and ***P < 0.001.

To determine whether Vitamin K2 is able to induce AMPK-dependent autophagic cell death *in vivo*, bladder cancer xenografts were established in nude mice. As shown in Fig. 8A, comparing with the sustained growth of tumor in vehicle group, treatment with Vitamin K2 at dose of 10 and 20 mg/kg gradually reduced the tumor sizes. Moreover, during drug administration, the weights of nude mice treated by Vitamin K2 were almost intact, compared with the significant decrease in vehicle group (Fig. 8B). Importantly, treatment with Vitamin K2 markedly extended the mice survival, compared with 100% death of vehicle group after administration for 58 days (Fig. 8C). To investigate whether the inhibitory growth effect of Vitamin K2 on bladder cancer cells *in vivo* is due to the induction of AMPK-dependent autophagic cell death, we excised the tumors from the mice, sectioned for immunohistochemistry (IHC) and lysed for western blot analysis. As shown in Fig. 8D, Vitamin K2 dose-dependently upregulated the expression of P62, Beclin-1 and LC3B II and activated Caspase-3 in tumor sections. Besides, the increased expression of GLUT-1, HIF-1α, p-AKT and p-AMPK were also detected in Vitamin K2-treated tumor group (Fig. 8E). Similar results were obtained from western blot analysis (Fig. 8F). Taken together, these results suggest that Vitamin K2 might promote the glycolysis and trigger AMPK-dependent autophagic cell death in bladder cancer cells *in vivo*.

## Discussion

This study was the first to show that Vitamin K2 could remarkably promote the glycolysis, whereas suppress TCA cycle in bladder cancer cells. Importantly, under glucose limited conditions, Vitamin K2-upregulated glycolysis could significantly induce metabolic stress in accompany with AMPK activation and mTORC1 pathway suppression, which subsequently triggers AMPK-dependent autophagic cell death in bladder cancer cells. In addition, following the inhibition of PI3K/AKT and HIF-1α or attenuation of glycolysis, the above phenotypes that Vitamin K2 induced metabolic stress and triggered AMPK-dependent autophagic cell death were markedly blocked, indicating that Vitamin K2 induces metabolic stress and triggers AMPK-dependent autophagic cell death via PI3K/AKT/HIF-1α activation and glycolysis promotion. In summary, the major novelty in this study is that Vitamin K2 is able to induce autophagic cell death in bladder cancer cells by establishing the metabolic signal axis: PI3K/AKT/HIF-1α dependent glycolysis promotion-metabolic stress-AMPK/ULK1 activation-mTORC1 pathway suppression (Fig. 8G).

Aerobic glycolysis is commonly hyper-activated in many cancer cells. The upregulated glycolysis in cancer cells profoundly provides rapid ATP generation and metabolic intermediates for the production of proteins, lipids and nucleotides, which meets the requirement for cell growth and metastasis. Traditional anticancer drugs are often reported to curb the growth of cancer cells via glycolysis blockade^34–37^. Different from the above anticancer mechanisms, our study showed that Vitamin K2 markedly promoted the glycolysis in bladder cancer cells, which induced metabolic stress and subsequently triggered AMPK-dependent autophagic cell death, upon glucose limitation. 2-DG, 3-BP and DCA are three typical glycolytic inhibitors that have been shown to exhibit excellent inhibitory growth effect on cancer cells by blocking the glycolytic pathway^38–40^. In this study, the above three glycolytic inhibitors were utilized to verify the hypothesis that Vitamin K2 induces metabolic stress and triggers AMPK-dependent autophagic cell death via glycolysis promotion. As expected, 2-DG, 3BP and DCA-induced glycolysis attenuation significantly prevented metabolic stress and rescued bladder cancer cells from Vitamin K2-triggered AMPK-dependent autophagic cell death, implying that Vitamin K2-increased glycolysis contributes to metabolic stress and AMPK-dependent autophagic cell death in bladder cancer cells.

Recent studies have revealed that hyper-activation of PI3K/AKT and HIF-1α in cancer cells plays a central role in glycolysis promotion. In our study, inhibition of PI3K/AKT and HIF-1α notably attenuated Vitamin K2-upregulated glycolysis, indicating that Vitamin K2 promotes glycolysis in bladder cancer cells via PI3K/AKT and HIF-1α signal pathways. Accumulating evidences suggest that Reactive oxygen species (ROS) is able to activate PI3K/AKT pathway by inactivating some protein phosphatase, such as protein tyrosine phosphatase 1B (PTP1B), protein phosphatase 2 A (PP2A) and phosphatase and tensin homologue (PTEN) lipid phosphatase^41^. In this study, we also attempt to investigate the involvement of ROS in Vitamin K2-activated PI3K/AKT pathway. As shown in Supplementary Fig. 7, N-acetyl cysteine (NAC, a ROS scavenger) not only alleviated Vitamin K2-induced AKT activation and glycolysis promotion, but also significantly suppressed the subsequent AMPK-dependent autophagic cell death. These results reveal that Vitamin K2-induced ROS generation activates AKT and subsequently promotes the glycolysis, resulting in AMPK-dependent autophagic cell death in bladder cancer cells.

Upon glucose limitation, the increased glycolysis will inevitably result in metabolic stress, which subsequently leads to cancer cell death. In our study, Vitamin K2-upregulated glycolysis significantly induced metabolic stress, including glucose starvation and energy shortage, and triggered AMPK-dependent autophagic cell death in bladder cancer cells. To validate whether Vitamin K2-induced metabolic stress results in the subsequent AMPK-dependent autophagic cell death, the glucose supplementation was performed. As shown in Fig. 5F–H, glucose supplementation not only restored c-MYC expression, but also rescued bladder cancer cells from Vitamin K2-triggered AMPK-dependent autophagic cell death, suggesting that metabolic stress induced by Vitamin K2 indeed triggers AMPK-dependent autophagic cell death in bladder cancer cells, under glucose limited condition.

AMPK is an energy sensing protein which is rapidly activated upon metabolic stress. Once activated, AMPK could repress the anabolism and simultaneously stimulate the catabolic process including autophagy to maintain intracellular energy homeostasis^16,42^. In line with the previous studies, our results showed that Vitamin K2 remarkably activated AMPK pathway, and triggered AMPK-dependent autophagy, in response to metabolic stress. Furthermore, apoptosis induced by Vitamin K2 was profoundly suppressed by respective treatment with CQ and 3MA, two typical autophagic inhibitors, suggesting that Vitamin K2-induced AMPK-dependent autophagy promotes the subsequent apoptosis in bladder cancer cells, upon metabolic stress. The mTORC1 pathway plays a pivotal role in the growth and survival of cancer cells by sensing intracellular nutrients levels. Upon metabolic stress, AMPK negatively regulates mTORC1 through phosphorylating TSC2 or Raptor (a mTORC1-positive regulatory subunit), resulting in suppression of mTORC1 pathway. In our study, inhibition of AMPK by AMPK siRNA or Compound C significantly reversed Vitamin K2-inhibited mTORC1, implying that AMPK activation contributes to mTORC1 pathway suppression in Vitamin K2-treated bladder cancer cells, upon metabolic stress.

Our previous studies indicated that Vitamin K2 is able to induce Mitochondria-related apoptosis in bladder cancer cells via ROS-JNK/P38 MAPK signal pathways. In that study, bladder cancer cells were directly exposed to Vitamin K2 for 24 hour, and a great amount of intracellular ROS generation was induced, which results in JNK/P38 MAPK activation and mitochondria-associated apoptotic cell death. By contrast, in this study, bladder cancer cells were time-dependently treated with Vitamin K2. At early stage, Vitamin K2 could promote glycolytic process in bladder cancer cells, which might prevent intracellular ROS accumulation. However, under glucose limited condition, the increased glycolysis inevitably resulted in metabolic stress, which augments ROS accumulation due to lack of glucose for sustained glycolysis. Therefore, we speculate that Vitamin K2-induced metabolic stress might trigger autophagy and apoptosis in bladder cancer cells by AMPK-ULK1 or ROS-JNK/P38 MAPK, two distinct signal pathways.

In conclusion, our study regarded Vitamin K2-upregulated glycolysis as a bridge to connect activation of PI3K/AKT and HIF-1α with metabolic stress, which triggers AMPK-dependent autophagic cell death in bladder cancer cells. Our study laid a solid foundation for unveiling the precise anticancer mechanism of Vitamin K2. In addition, the anticancer function of Vitamin K2 through glucose metabolic regulation might shed insight into starving cancer cells in the real tumor microenvironment.

## Materials and Methods

### Reagents

Vitamin K2 (V9378), α-D-Glucose (158968), 2-Deoxy-D-glucose (D8375), 3-Bromopyruvic acid (16490) and Sodium dichloroacetate (347795) were purchased from Sigma-Aldrich. Lificiguat (YC-1) (S7958) and Compound C (S7840) were purchased from Selleck. MK2206 dihydrochloride (HY-10358), LY294002 (HY-10108), Chloroquine (HY-17589), 3-Methyladenine (HY-19312) and Rapamycin (HY-10219) were purchased from MCE.

### Cell culture

Human bladder cancer T24, EJ and J82 cells were obtained from the American Type Culture Collection (Manassas, VA, USA) and cultured in Minimum Essential Medium Eagle (MEM) (SH30024.01, Hyclone) supplemented with 10% Fetal Bovine Serum (11011-8611, Every Green). Cell cultures were maintained at 37 °C in a humidified atmosphere of 5% CO_2_ incubator.

### Glucose consumption and lactate production assays

Cells were seeded at a density of approximately 1×10^6^ cells per well, and treated with Vitamin K2 for the indicated time. The glucose consumption and lactate production were respectively assessed using the glucose and lactate content detecting kits (E1010, Applygen) (A019-2, Nanjing Jiancheng) according to the manufacturer’s protocols, and measured by microplate readers.

### Liquid chromatography–mass spectrometry (LC-MS) assays

T24 cells were seeded in 10cm-cell culture dishes and treated with 50 μM Vitamin K2 for the indicated time. For cellular metabolite extracting, T24 cells were washed with PBS and fixed with the metabolite extraction buffer consisting of methanol, acetonitrile, and water (2:2:1). Cells were then scraped and transferred into 1.5 ml centrifuge tubes and vortexed for 30 seconds. Samples were centrifuged at 14,000 × g and the supernatants were harvested for analysis. The metabolites were analyzed by LC-MS using a Agilent 1290 Infinity LC system coupled to a 5500 QTRAP mass spectrometer (AB SCIEX). Briefly, 4 μl of sample was injected on a column and heated at 45°C. The flow rate was set to 300 μl/min. The gradient mobile phase was composed by water with 15 mM CH3CO2NH4 (A) and acetonitrile (B). The program with a linear gradient beginning at 90% (B) and decreasing to 40% (B) for 18 min was used and followed by washing and re-equilibration. The total running time is 23 min. The metabolites were analyzed by a 5500 QTRAP mass spectrometer under the anion mode conditions. The ion pairs were detected in the mode of MRM. The data processing was performed using Multiquant software.

### Western blot

Cells were seeded in 6-well plates at a density of approximately 1×10^6^ cells per well. Following treatment with Vitamin K2 combined with or without the correlative inhibitors or siRNA, cells were washed with PBS, then protein lysates were prepared by the RIPA buffer (Beyotime, China) supplemented with protease inhibitor cocktail (Google Biology, Wuhan). After 30 minutes lysis, sample lysates were centrifuged at 12,000 × g for 15 minutes, then obtained the supernatants. The total protein were separated by SDS-PAGE and transferred to PVDF membranes (ISEQ. 00010, Millipore). The membranes were subsequently blocked with 5% fat-free milk and probed with the primary antibodies for overnight at 4°C, and secondary antibodies for 2 hours at room temperature. The protein expression was determined using a chemiluminescence solution (ECL) (34094, Thermo Fisher). Western blot was performed with the following antibodies: anti-Glut-1 (21829-1-AP, Proteintech), anti-HexoKinase II (2867, Cell Signaling Technology), anti-PFKFB2 (A9311, ABclonal), anti-LDHA (ab101562, Abcam), anti-PDHK1 (3820, Cell Signaling Technology), anti-GAPDH (ABM40040, Abbkine), anti-p-PDH E1α (ser293) (ab177461, Abcam), anti-p-PI3K (abs130868, Absin Bioscience), anti-p-AKT (ser473) (9271, Cell Signaling Technology), anti-c-MYC (ab32072, Abcam), anti-AKT (ab188099, Abcam), anti-HIF-1α (ab51608, Abcam), anti-p-AMPKα (Thr172) (2535, Cell Signaling Technology), anti-AMPKα (ab32047, Abcam), anti-p-ACC (ab68191, Abcam), anti-p-mTOR(ser2448) (5536, Cell Signaling Technology), anti-p-P70S6K(Thr389) (9234, Cell Signaling Technology), anti-p-ULK1(ser555) (5869, Cell Signaling Technology), anti-P62 (5114, Cell Signaling Technology), anti-Beclin-1 (3495, Cell Signaling Technology), anti-LC3B (ab192890, Abcam), anti-Caspase-9 (PB0285, Boster), anti-Caspase-3 (9662, Cell Signaling Technology). All the western blots were repeated at least three times, and the density of each band was measured by Quantity One software, and density value including mean and standard deviation between replicates was determined by GraphPad Prism 6 software.

### Immunofluorescence

Cells were seeded in 6-well plates, and grown on the coverslips. After treatment with 50 μM Vitamin K2 for 14 hours, cells were washed with PBS and fixed with 4% paraformaldehyde, then incubated with LC3 primary antibody at 4°C for overnight, and secondary antibodies for 2 hours. The intracellular LC3 expression was observed under the fluorescence microscope (Olympus, Japan).

### Electron microscopy

Cells were treated with 50 μM Vitamin K2 for 14 hours, washed with PBS, trypsinized, and collected. The cell pellets were fixed with 4% glutaraldehyde, embedded in the paraffin, and sectioned. The autophagosomes were observed under the transmission electron microscope (Olympus, Japan).

### Cell viability analysis

Cells were seeded in 96-well plates at a density of approximately 1×10^5^ cells per well, and treated by 50 μM Vitamin K2 combined with or without the correlative inhibitors for 18 hours. The cell viability was evaluated by the MTS assays in accordance with the manufacturer’s protocol (G3580, Promega).

### Apoptosis analysis

Cells were seeded in 12-well plates at a density of approximately 1×10^6^ cells per well, and treated by 50 μM Vitamin K2 combined with or without the correlative inhibitors for 18 hours. To determine cell apoptosis, cells were collected, washed with PBS, and suspended in binding buffer. Cells were then stained with Annexin V-FITC and propidium iodide (PI) according to the instructions of an Annexin-V-FITC/PI apoptosis detecting kits (556547, BD Biosciences). The apoptotic cells were analyzed by the flow cytometer (Beckman coulter FC500).

### SiRNA transfection

The siRNA targeting Human HIF-1α, AMPK, AKT and PI3K were synthesized by the Gene Pharma Company (Suzhou, China). The relevant sequences are as follows: siHIF-1α,5′-GGAUGCUGGUGAUUUGGAUAUUGAAdTdT-3′, siAMPK, 5′-CAAAUGCUUCCAUUUGUAA-3′, siAKT, 5′-CUCACAGCCCUGAAGUACUtt-3′, siPI3K, 5′-GGUGAAAGACGAUGGACAA-3′ andsiControl, 5′-UUCUCCGAACGUGUCACGUTT-3′. Briefly, cells were seeded in 6-well plates at a density of approximately 1×10^5^ cells per well. After incubating for 24 hours, cells were transfected by siRNA using lipofectamine 2000 (11668019, Invitrogen). Following 72 hours interference, cells were treated with 50 μM Vitamin K2 for the indicated time.

### Animal experiments

Female BALB/c nude mice (4–5 weeks old) were provided by experimental animal center (Tongji Medical college of Huazhong University of Science and Technology). All animal studies were strictly conducted in compliance with guidelines approved by both the Science and Technology Department of Hubei province and the Animal Experimentation Ethics Committee of Huazhong University of Science and Technology. Bladder cancer EJ cells (1×10^7^) were subcutaneously injected into the right flank of nude mice. When the tumor sizes reached approximately 100 mm^3^, the tumor-bearing nude mice were intra-tumorally treated with 10 and 20 mg/kg Vitamin K2 each day, while intra-tumor injection of the equivalent volume of PBS as the vehicle group. For evaluating the changes of tumor sizes, the sliding caliper was used and the tumor sizes were measured by the formula: length × width^2^/2. For detecting the expression of the relevant proteins, mice were sacrificed and the tumors were excised, and lysed for western blot or sectioned for immunohistochemistry (IHC). Specific explanation for the absence of image of adequate length (Fig. 8F): *In vivo* experiment of western blot, the gels containing the separated protein were cropped, then transferred in PVDF, incubated with antibodies, and exposed in the films. Therefore, no strict fuller-length of gels was provided in our Supplementary information file, only the original cropped films with labels were displayed.

### Statistical analysis

The results were presented as the Mean ± SD for representative experiments with at least three time independent biological repeats, and analyzed using the GraphPad Prism 6 software. The statistical significance was measured using Student’s t-test. The statistical significance in the figures was set at *p < 0.05, **p < 0.01, ***p < 0.001, ns: no significance.

## Supplementary information

Supplementary information.
