# Supplementary material for: Vitamin K2 promotes PI3K/AKT/HIF-1α-mediated glycolysis that leads to AMPK-dependent autophagic cell death in bladder cancer cells
Source: Sci Rep. 2020 May 7;10:7714. doi: 10.1038/s41598-020-64880-x (PMC7206016; doi:10.1038/s41598-020-64880-x)

Supplementary information

Vitamin K2 promotes PI3K/AKT/HIF-1α-mediated glycolysis that leads to AMPK-dependent autophagic cell death in bladder cancer cells

Fengsen Duan[1], Chunlei Mei[2], Luhao Yang[2], Junyan Zheng[2], Huiai Lu[1], Yanzhi Xia[1], Stacy Hsu[4], Huageng Liang[3*] and Ling Hong[1*]

[1] Department of Biology, College of Life Science and Technology, Huazhong University of Science and Technology, 430074 Wuhan, China

[2] Tongji Medical College, Huazhong University of Science and Technology, 430030 Wuhan, China

[3] Department of Urology, Union Hospital, Tongji Medical College, Huazhong University of Science and Technology, 430022 Wuhan, China

[4] Global Public Health (Biology), Pre-Medicine Track, New York University College of Arts& Science 2021, 10003 New York, USA

*Correspondence: Huageng Liang (leonard19800318@hust.edu.cn)

Ling Hong (lhong@mail.hust.edu.cn)

Supplementary Figure 1

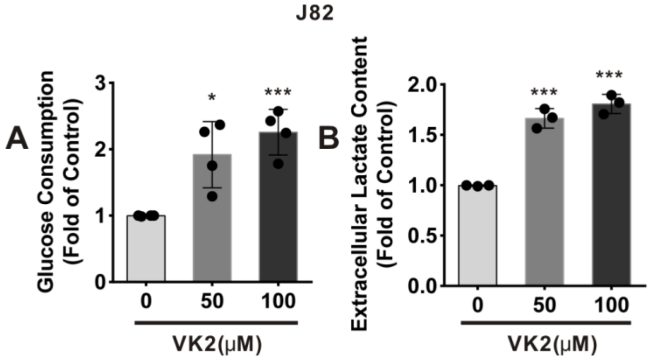

Supplementary Figure 1 Vitamin K2 increases glucose consumption and lactate production in bladder cancer J82 cells. (A) Vitamin K2 dose dependently affected glucose consumption in J82 cells. (B) Vitamin K2 dose dependently affected lactate generation in J82 cells. Data are presented as the mean ± SD of three or four independent experiments. * $P<0.05$ and *** $P<0.001$.

Supplementary Figure 2

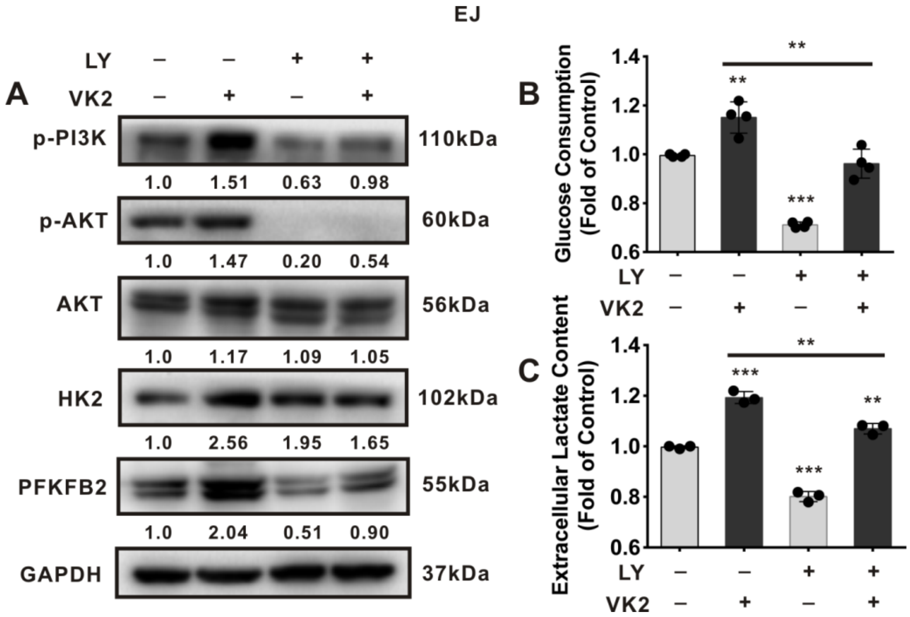

Supplementary Figure 2 Activation of PI3K/AKT is involved in Vitamin K2-induced upregulation of glucose consumption and lactate generation in bladder cancer EJ cells. (A) The effect of 40μM LY294002 (LY, a typical PI3K inhibitor) on the activation of PI3K and AKT as well as the expression of HK2 and PFKFB2 in EJ cells treated with or without 50μM Vitamin K2. (B) and (C) The effect of LY294002 on the glucose consumption and lactate production in EJ cells treated with or without Vitamin K2. Data are presented as the mean ± SD of three or four independent experiments. ** P<0.01 and *** P<0.001.

Supplementary Figure 3

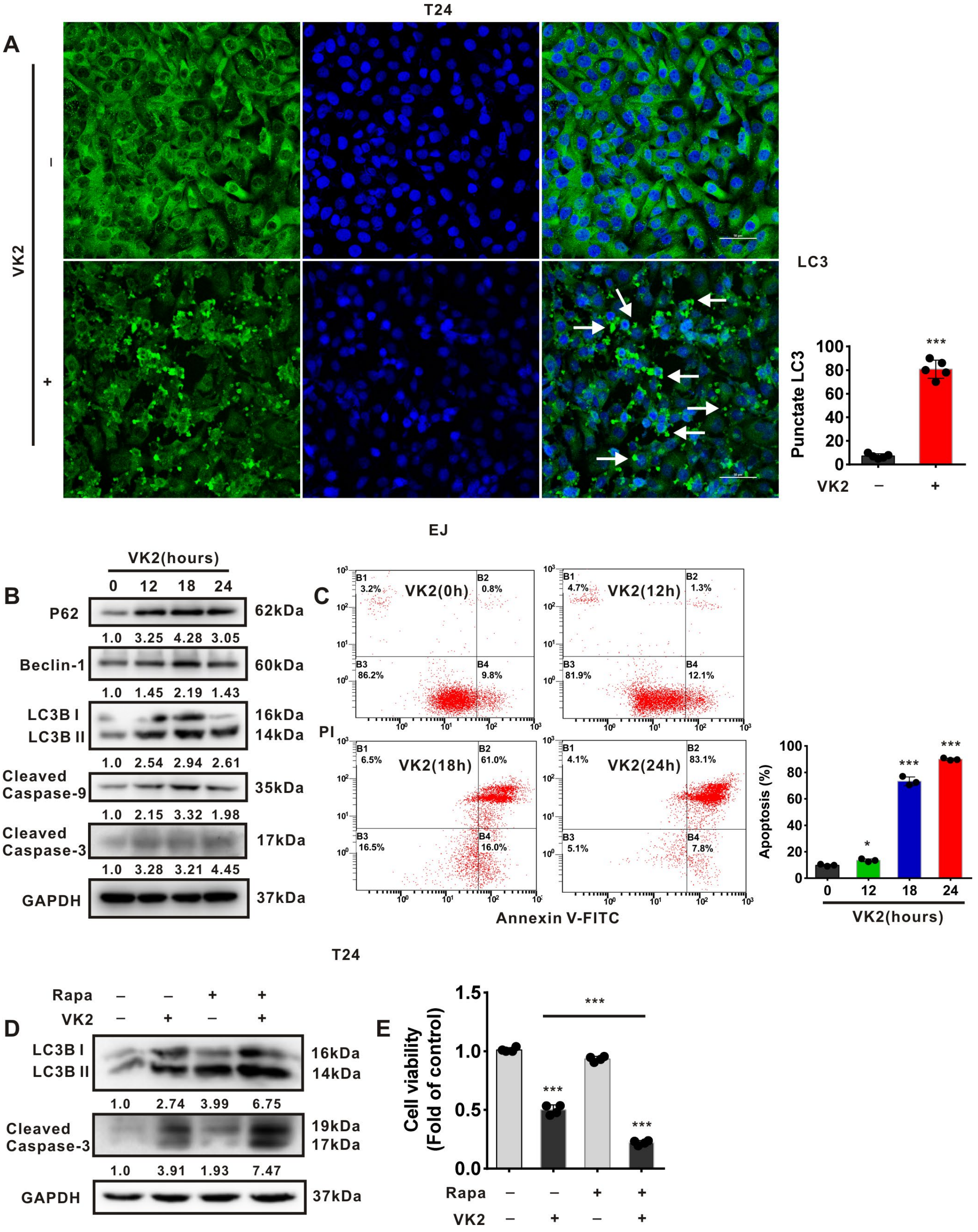

Supplementary Figure 3 Vitamin K2 induces autophagy and apoptosis in bladder cancer cells. (A) Immuno-Fluorescence demonstrated the expression of LC3 in T24 cells treated with or without 50μM Vitamin K2 for 18 hours. Arrow pointed to

punctate LC3. Scale bar: 50μm. (B) Western blot demonstrated the effect of Vitamin K2 on the expression of autophagic and apoptotic protein in EJ cells. (C) Flow cytometry demonstrated the apoptotic effect of Vitamin K2 on EJ cells in a time dependent manner. (D) Western blot demonstrated the effect of 20μM Rapamycin on Vitamin K2-induced autophagy and apoptosis in bladder cancer T24 cells. (E) MTS assays showed the effect of Rapamycin on the cell viability of T24 cells treated with or without 50μM Vitamin K2. Data are presented as the mean ± SD of three or four independent experiments. * $P<0.05$ and *** $P<0.001$.

Supplementary Figure 4

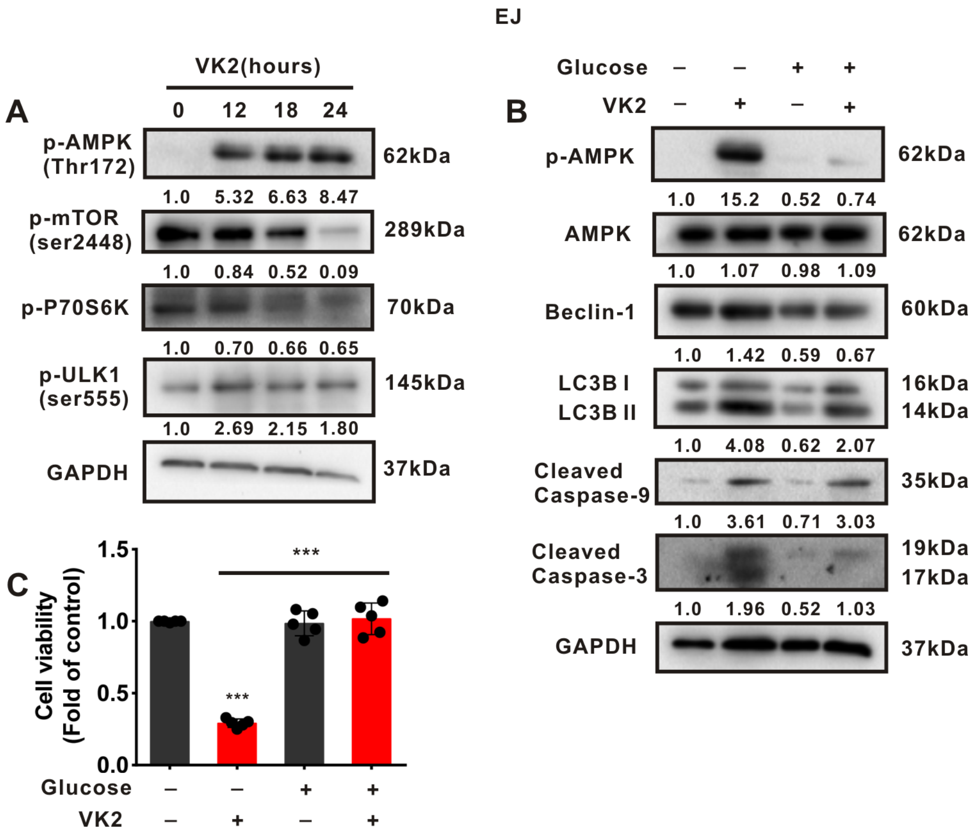

Supplementary Figure 4 Glucose supplementation rescues bladder cancer EJ cells from AMPK-dependent autophagic cell death. (A) Western blot showed the effect of Vitamin K2 on the activation of AMPK and mTORC1 pathway in bladder cancer EJ cells. (B) Western blot showed that 10mM glucose supplementation affected Vitamin K2-triggered AMPK activation, autophagy and apoptosis in EJ cells. (C) MTS assays showed that glucose supplementation affected the cell viability of EJ cells treated with or without 50μM Vitamin K2. Data are presented as the mean ± SD of three or four independent experiments. *** P<0.001.

Supplementary Figure 5

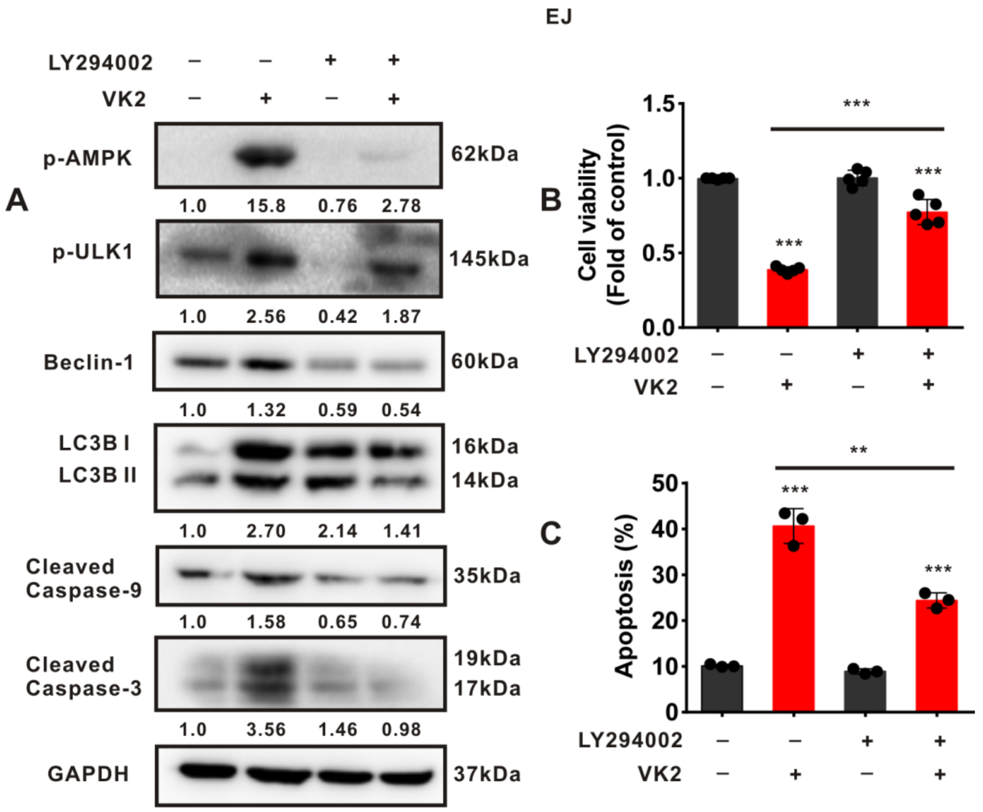

Supplementary Figure 5 Activation of PI3K/AKT is required for Vitamin K2-triggered AMPK dependent autophagic cell death in bladder cancer EJ cells. (A) Western blot showed the effect of 40μM LY294002 (LY, a typical PI3K inhibitor) on AMPK activation, autophagy and apoptosis in Vitamin K2-treated EJ cells. (B) MTS assays showed the effect of LY294002 on EJ cell viability after treatment with or without 50μM Vitamin K2. (C) Flow cytometry showed the effect of LY294002 on Vitamin K2-induced apoptosis in EJ cells. Data are presented as the mean ± SD of at leat three independent experiments. ** $P<0.01$ and *** $P<0.001$.

Supplementary Figure 6

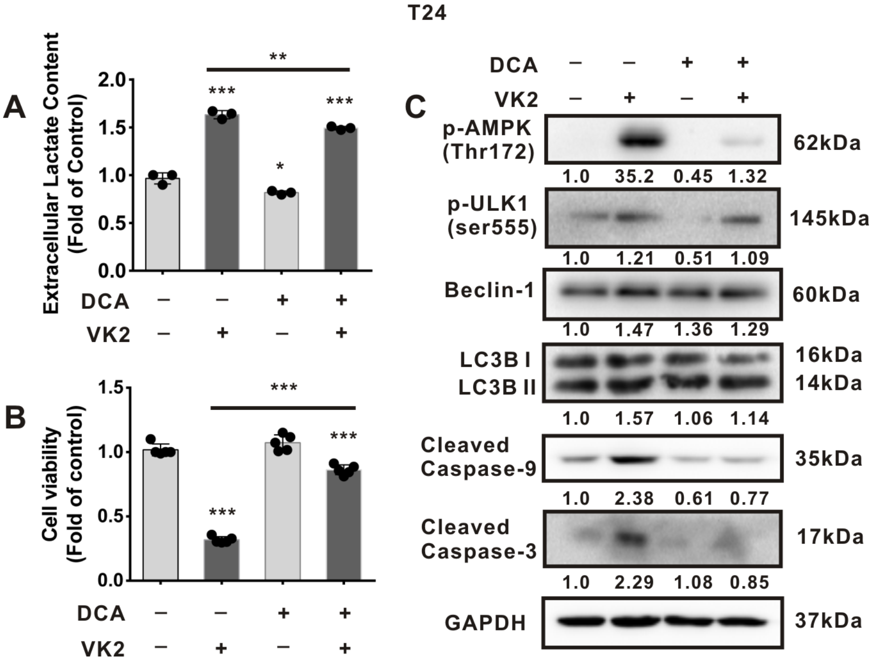

Supplementary Figure 6 DCA attenuates the glycolysis and blocks Vitamin K2-induced AMPK dependent autophagic cell death in bladder cancer T24 cells. (A) The effect of 30mM DCA on the lactate generation in T24 cells treated with or without 100μM Vitamin K2. (B) The effect of DCA on the cell viability of T24 cells treated with or without Vitamin K2. (C) Western blot showed that DCA affected Vitamin K2-induced AMPK dependent autophagic cell death in T24 cells. Data are presented as the mean ± SD of three or four independent experiments. * P<0.05, ** P<0.01 and *** P<0.001.

Supplementary Figure 7

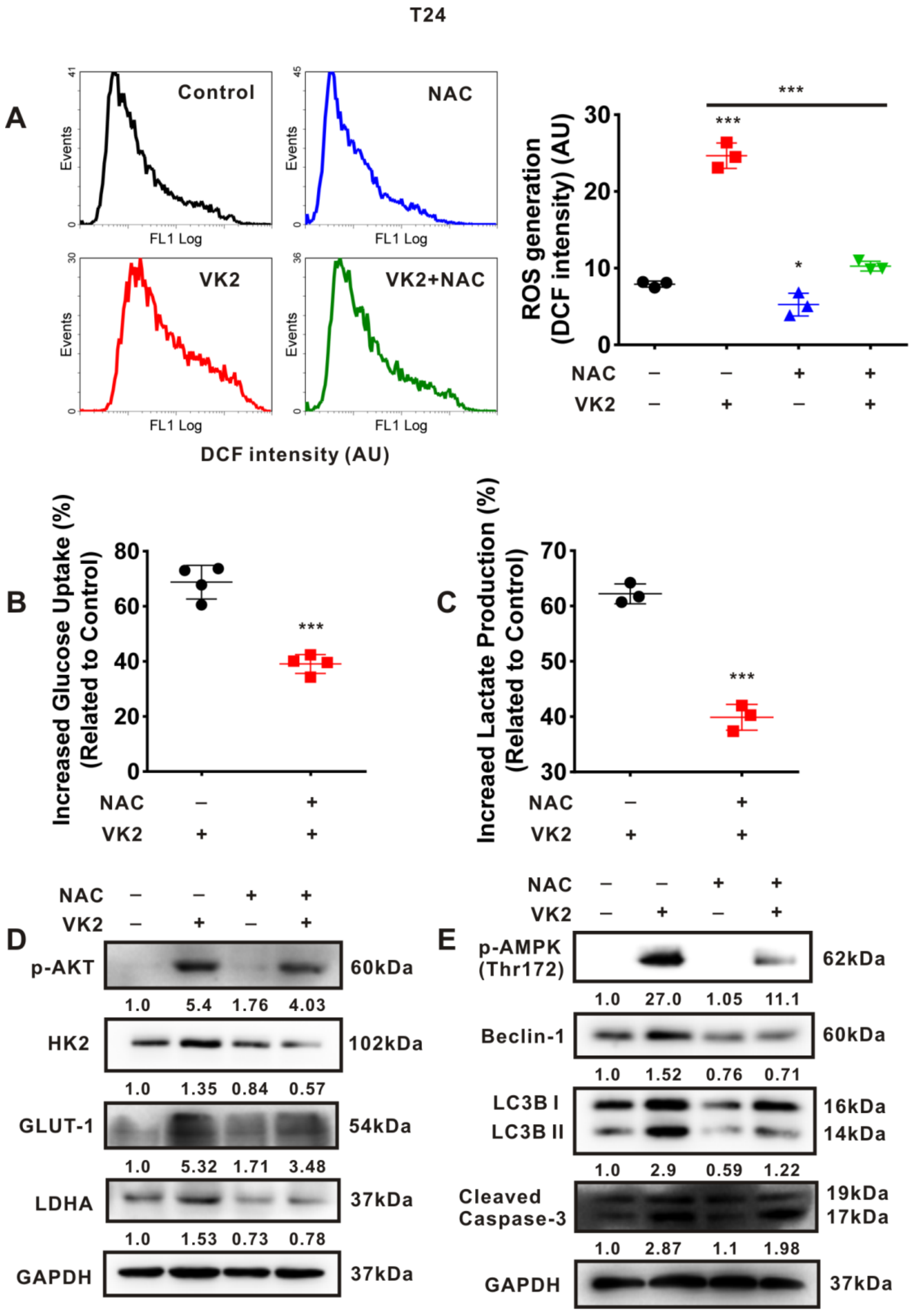

Supplementary Figure 7 ROS generation is required for Vitamin K2-upregulated glycolysis and triggered AMPK-dependent autophagic cell death in bladder

cancer T24 cells. (A) Flow cytometry showed the effect of 5mM N-acetyl cysteine (NAC, a ROS scavenger) on Vitamin K2-induced ROS generation in bladder cancer T24 cells. (B) and (C) The effect of NAC on Vitamin K2-elevated glucose uptake and lactate production in bladder cancer T24 cells. (D) Western blot showed the effect of NAC on the expression of glycolytic protein or enzymes in T24 cells treated with or without 50μM Vitamin K2. (E) Western blot showed the effect of NAC on AMPK activation, autophagy and apoptosis in T24 cells treated with or without Vitamin K2. Data are presented as the mean ± SD of three or four independent experiments. * $P<0.05$ and *** $P<0.001$.

The original unprocessed images for western blot and the target panels were marked in red boxes.

T24

Figure 1E

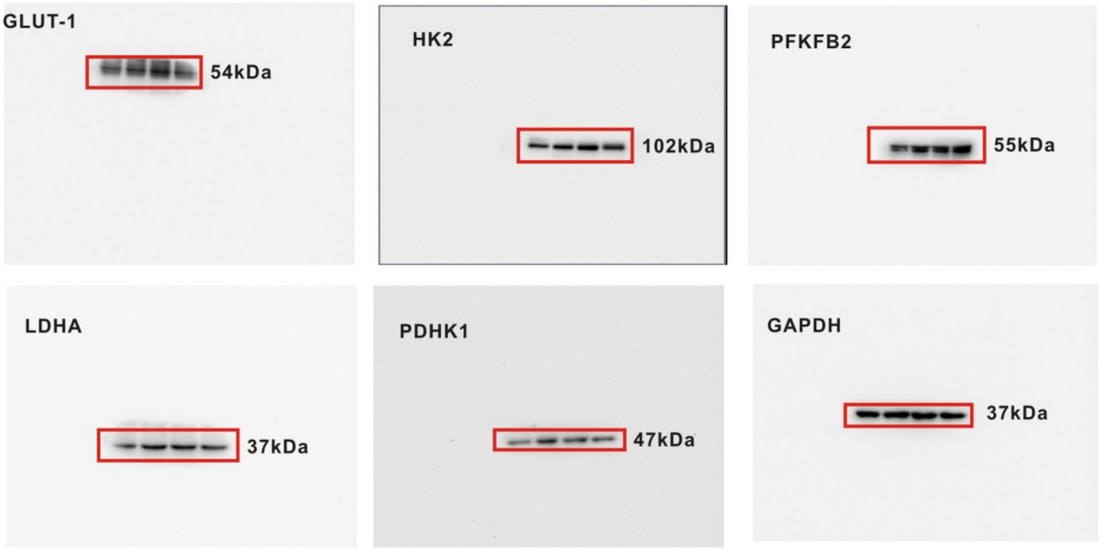

EJ

Figure 1F

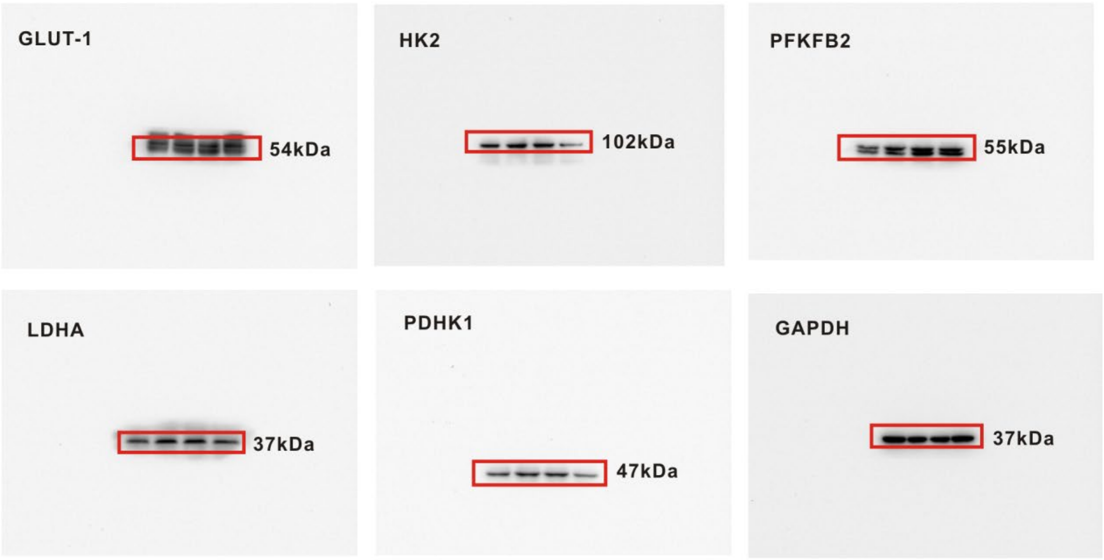

Figure 1H

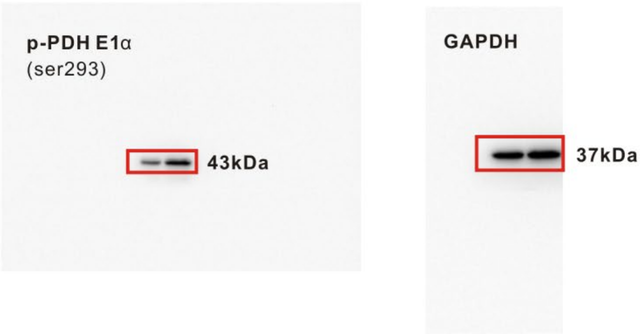

Figure 2A

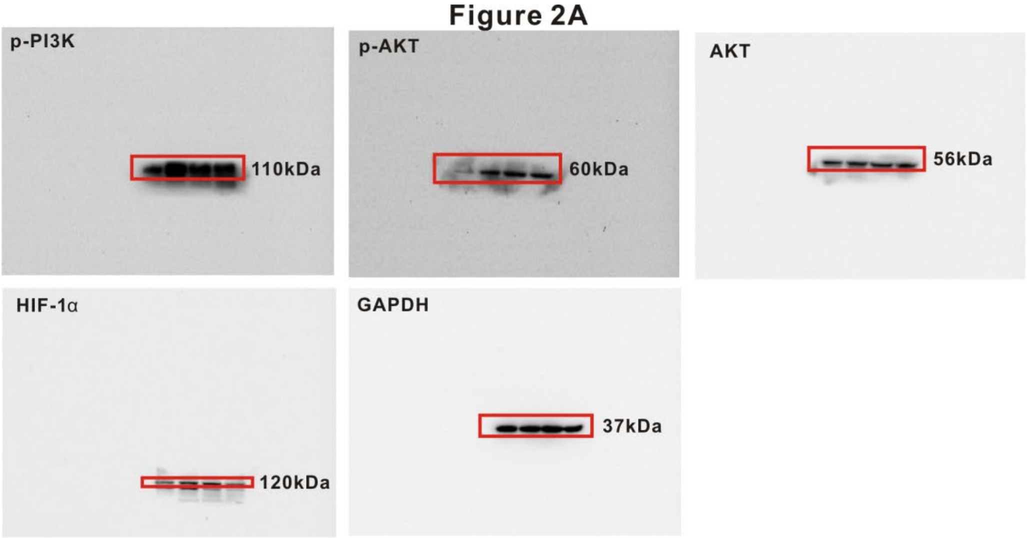

Figure 2B

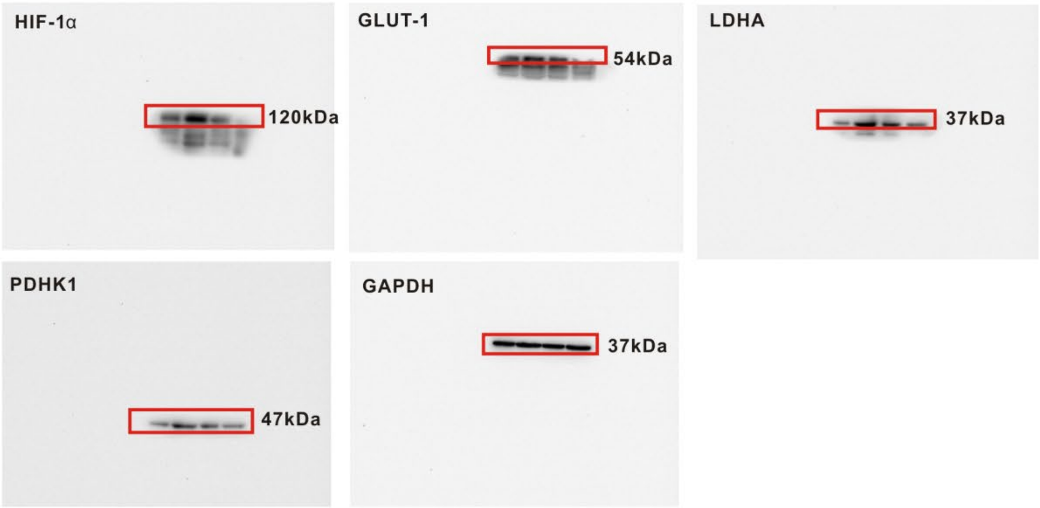

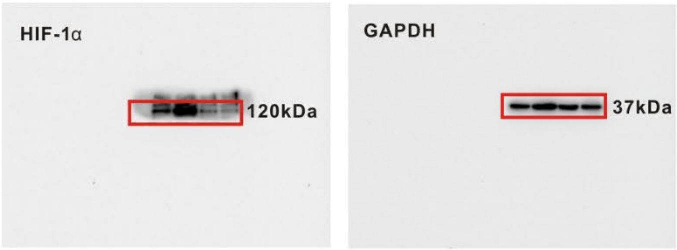

Figure 2G

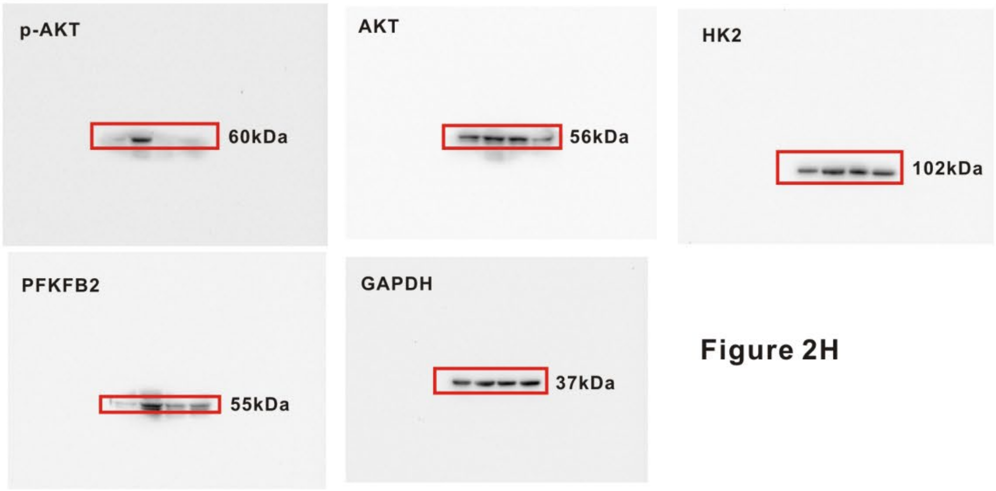

Figure 2H

Figure 2K

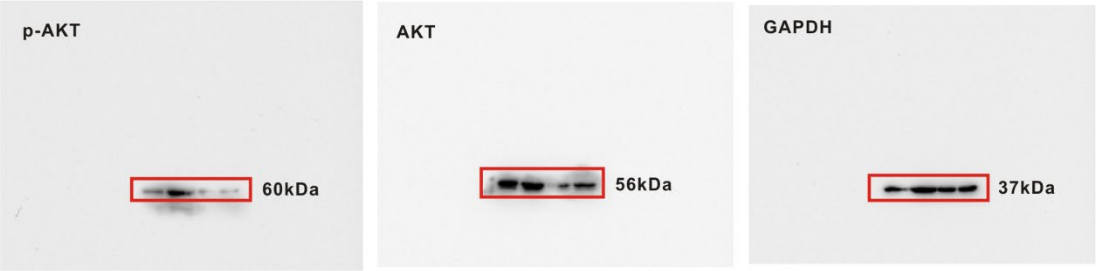

Figure 2N

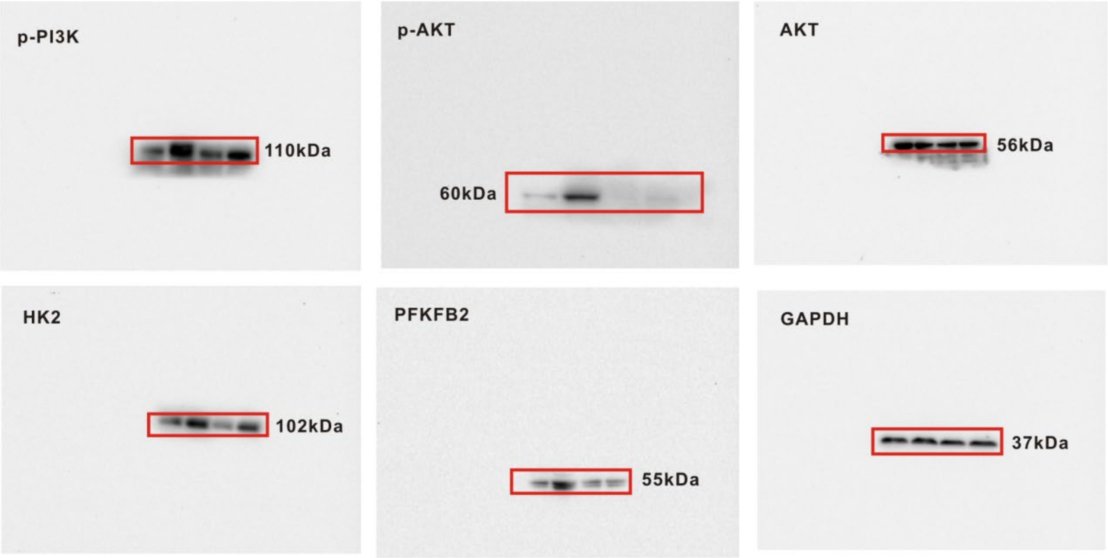

Figure 2Q

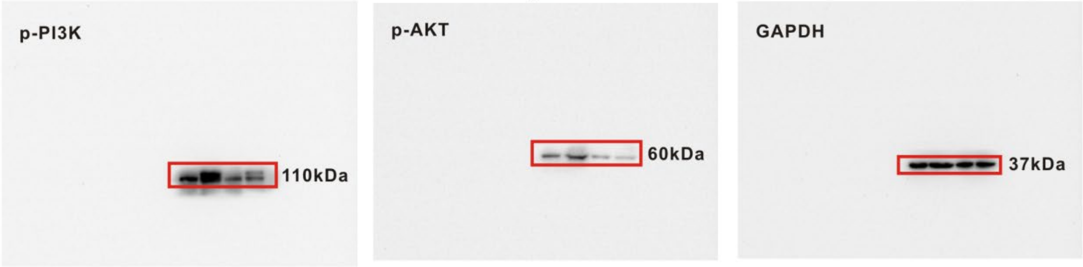

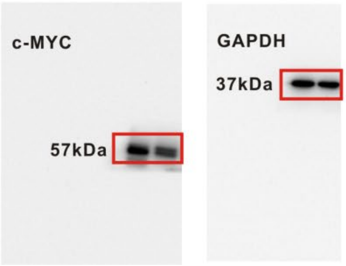

Figure 3D

Figure 4A

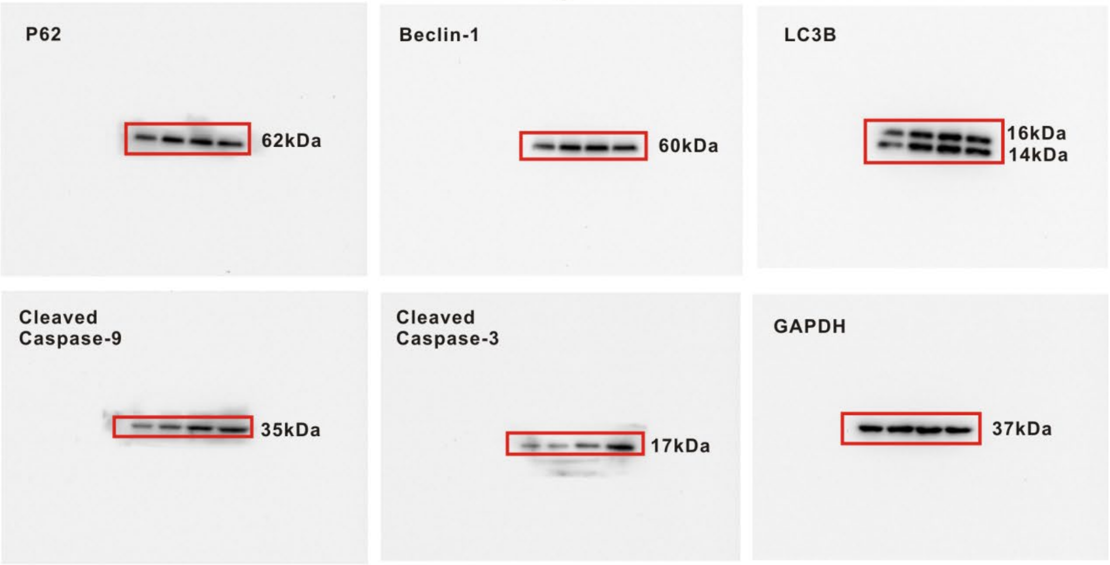

Figure 4D

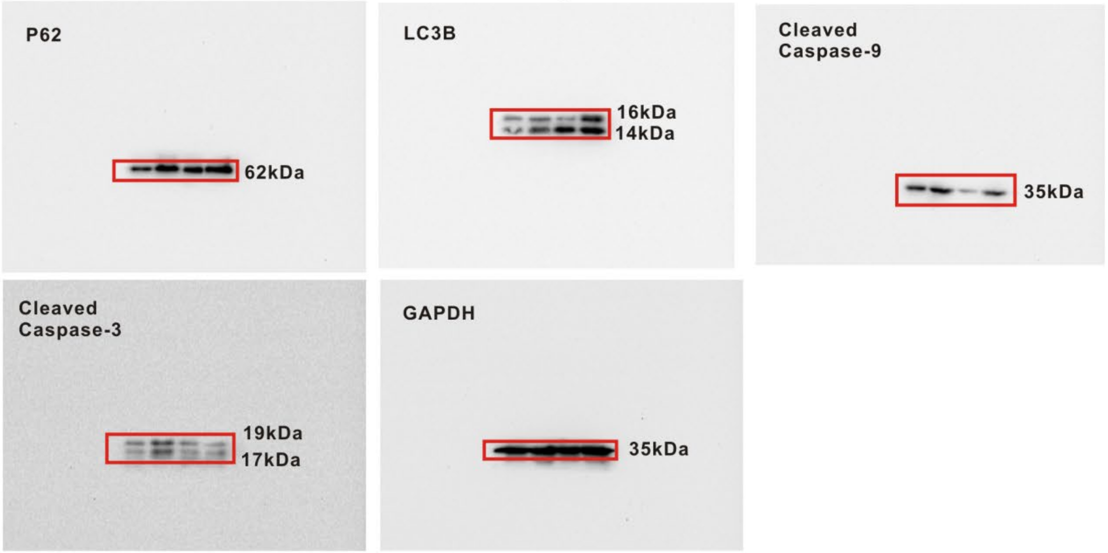

Figure 4E

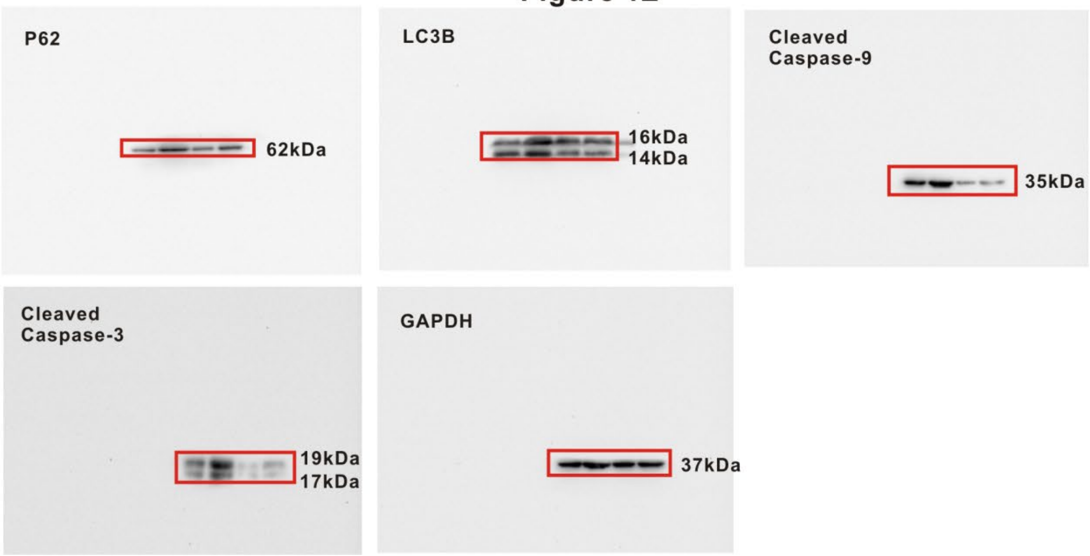

Figure 5A

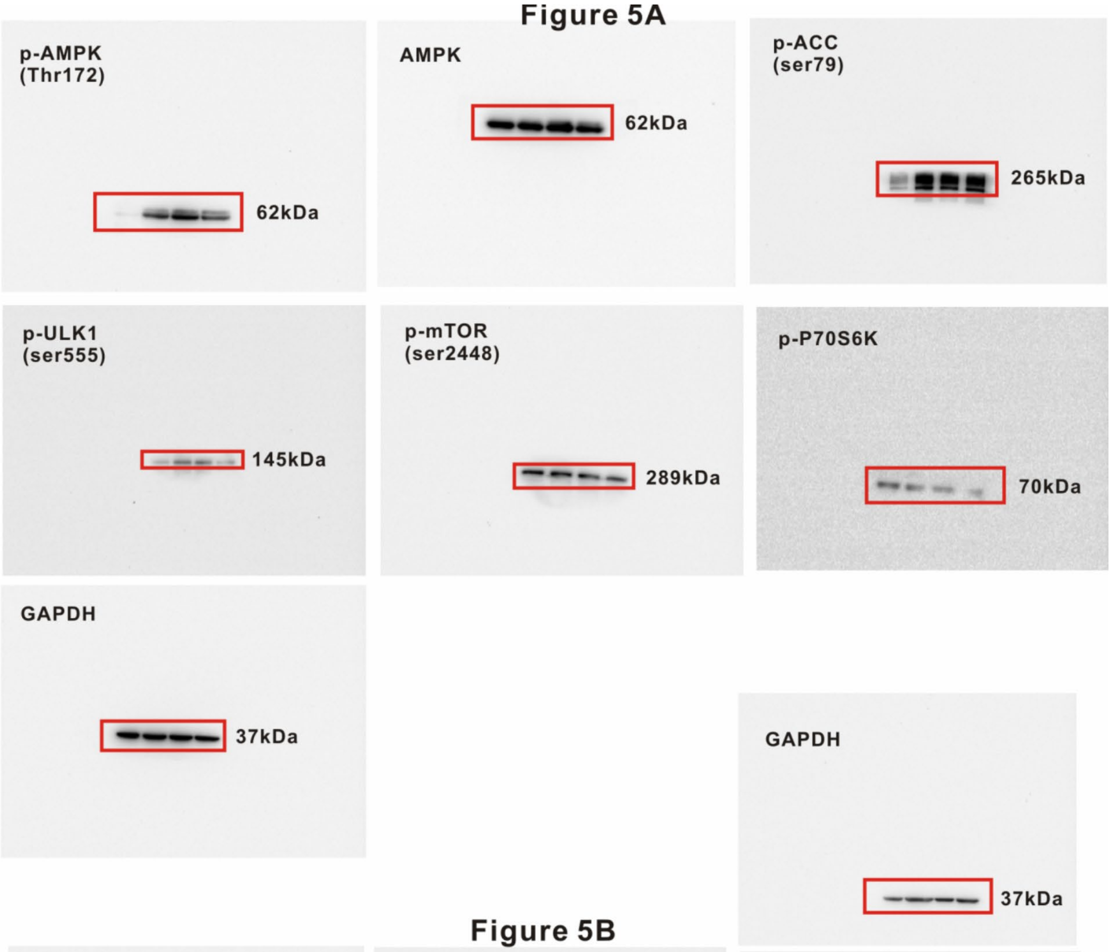

Figure 5B

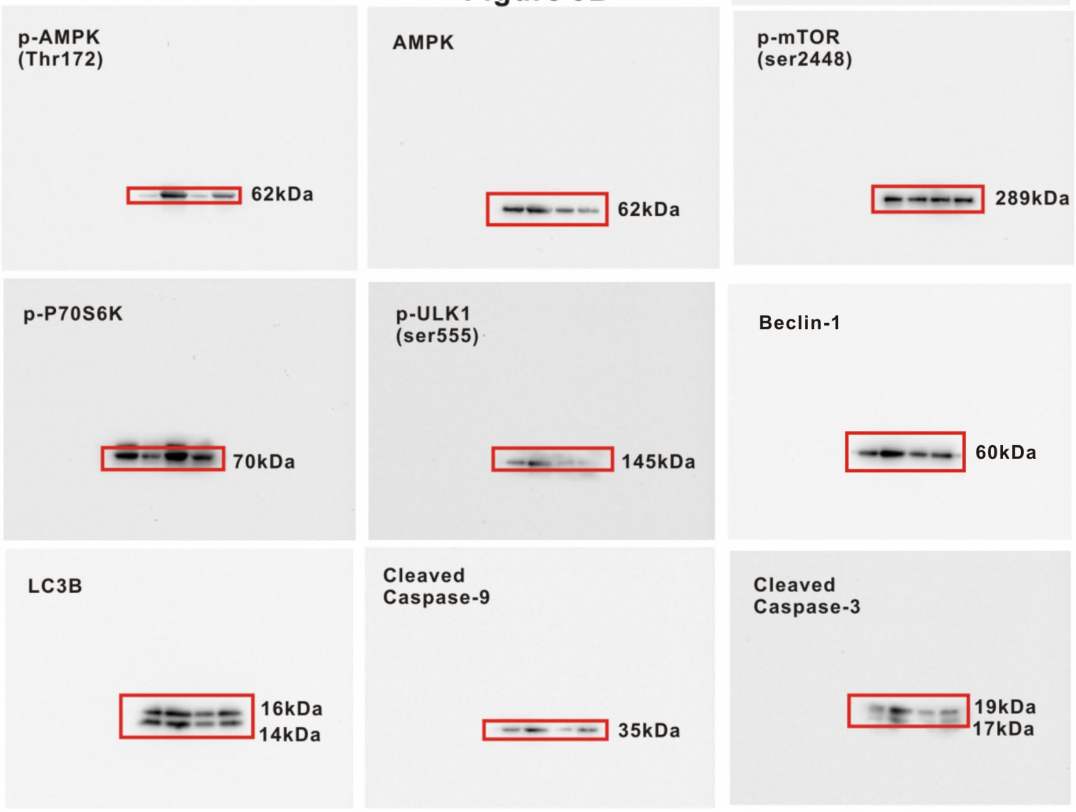

Figure 5C

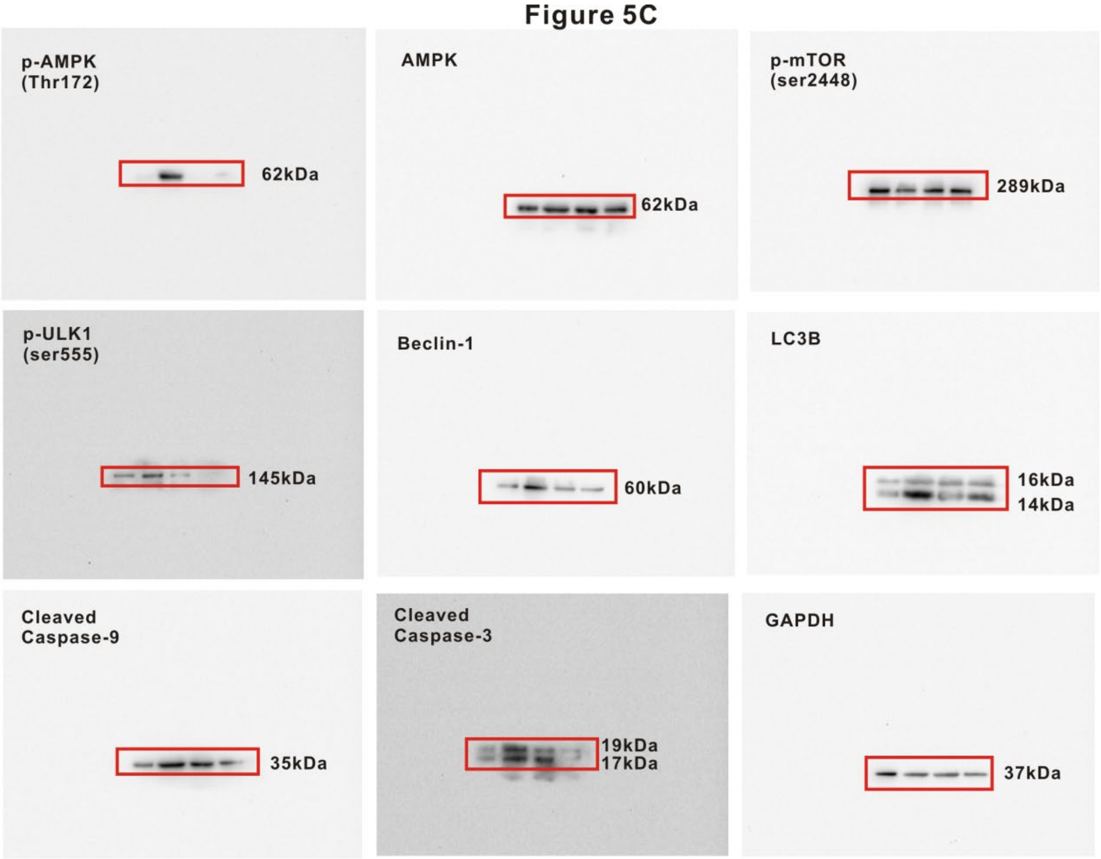

Figure 5F

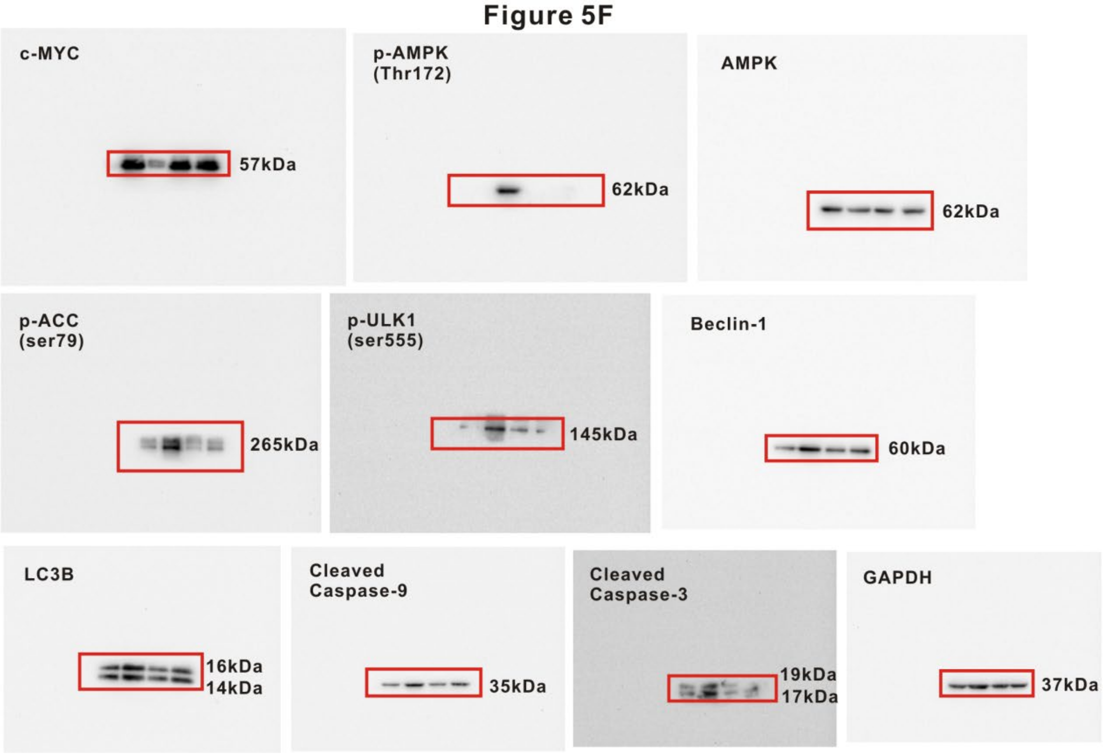

Figure 6A

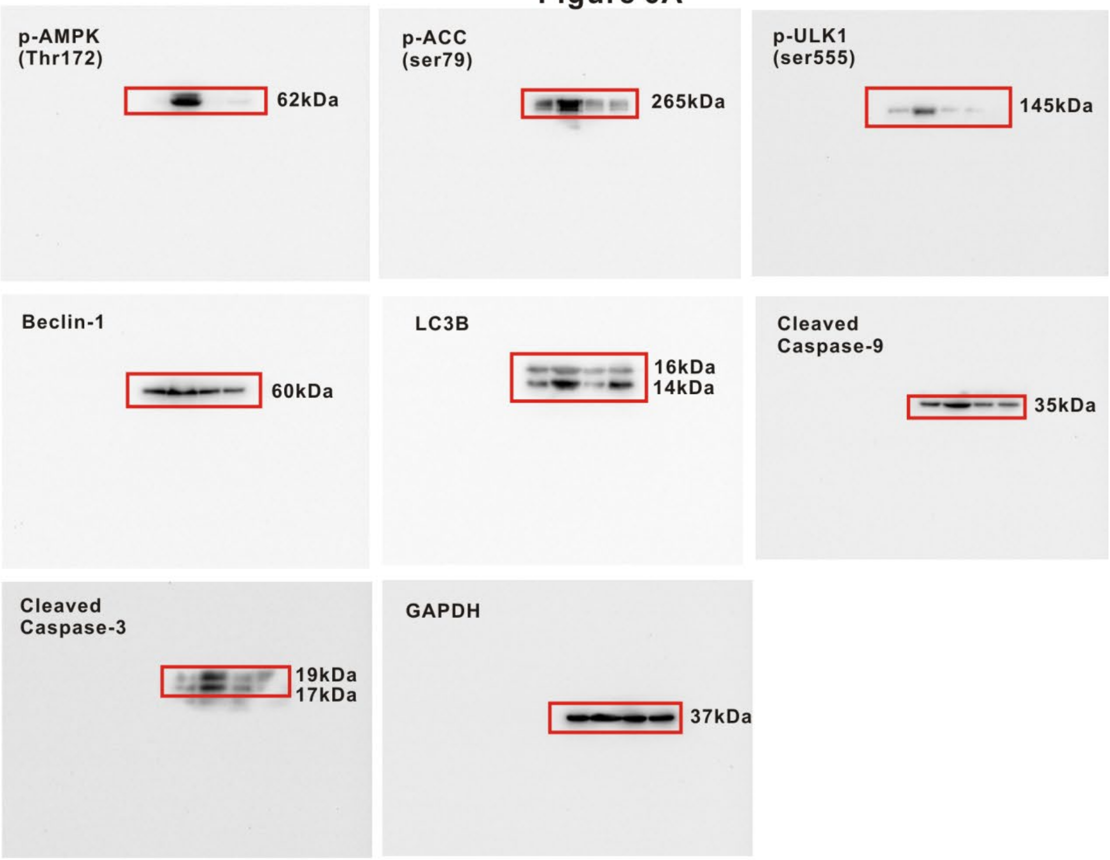

Figure 6B

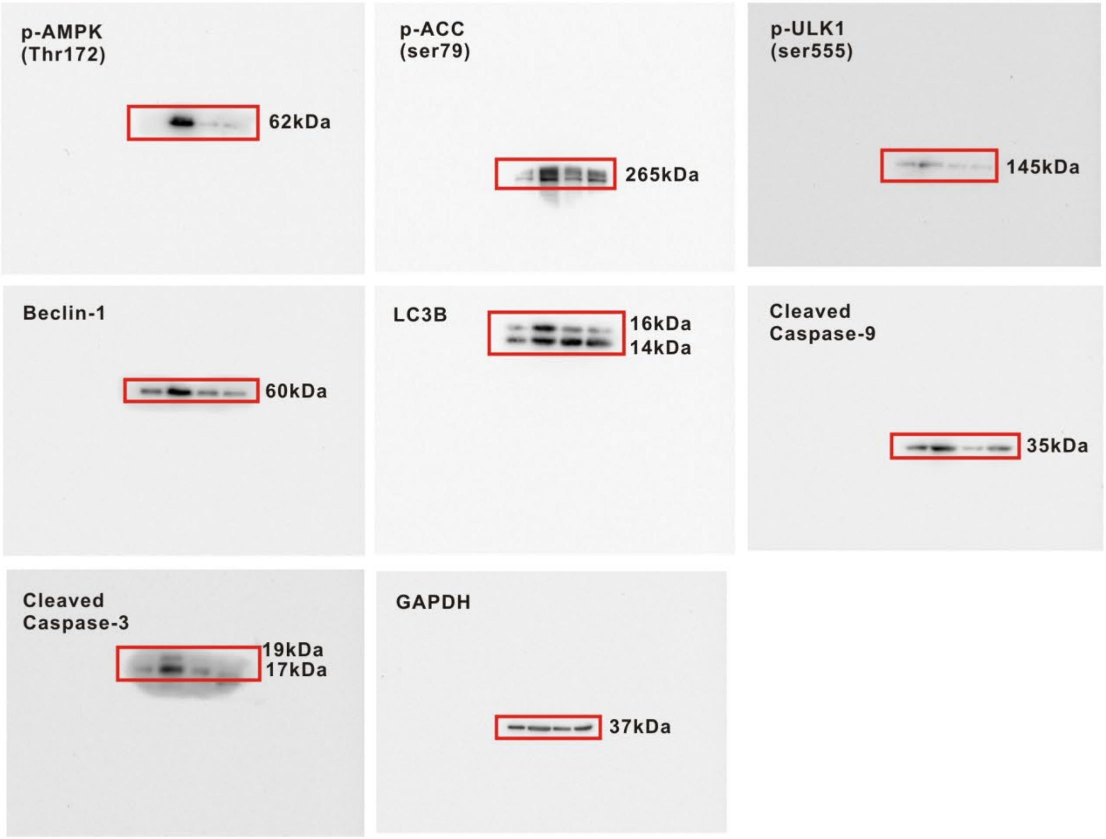

Figure 6E

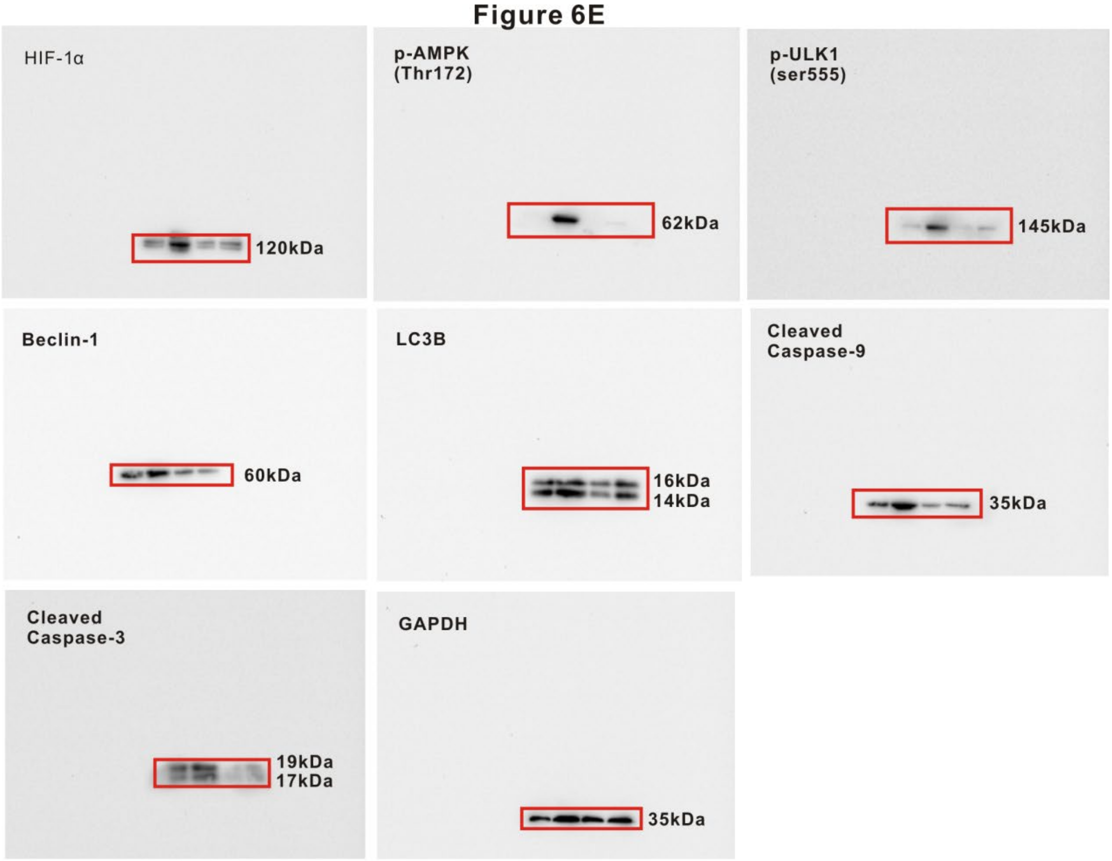

Figure 6F

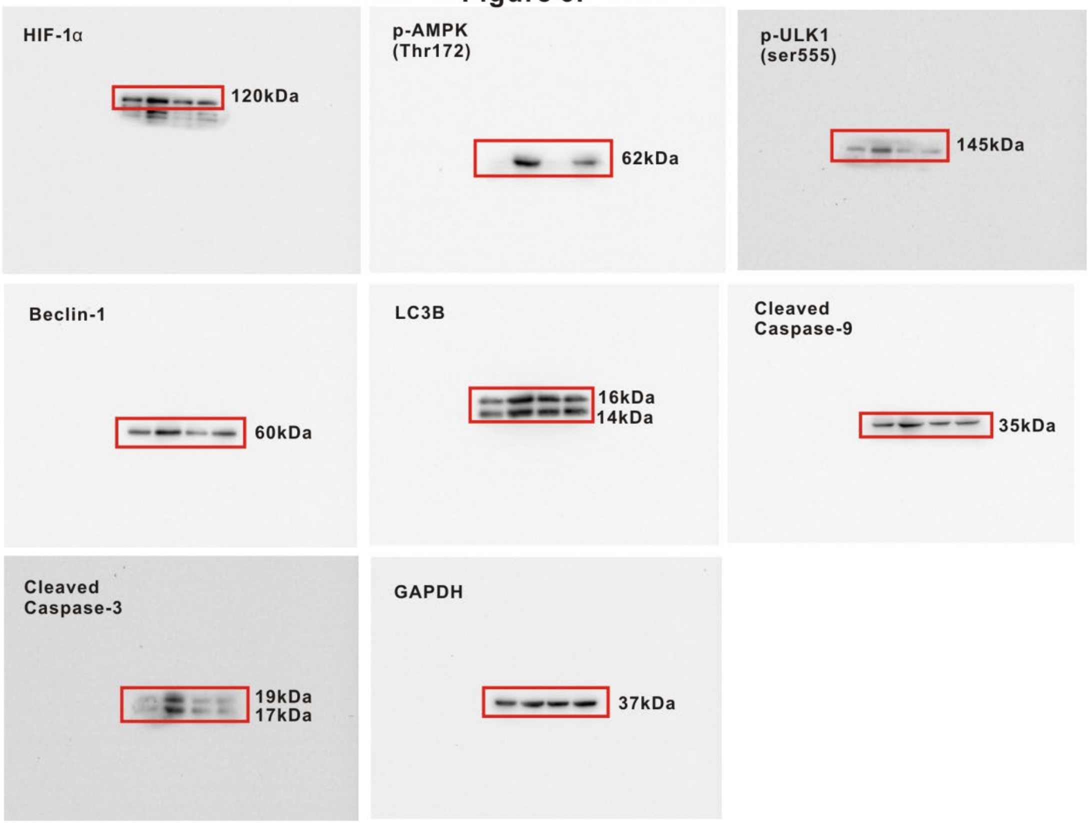

Figure 6I

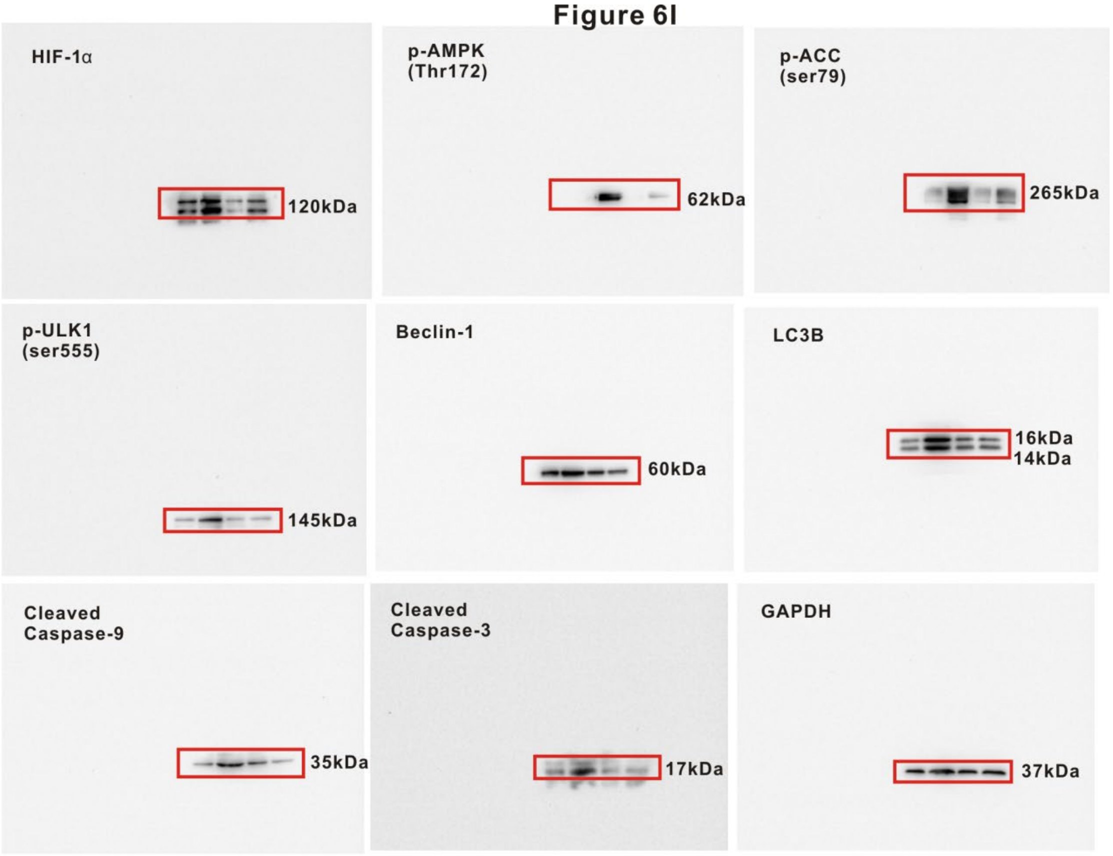

Figure 6J

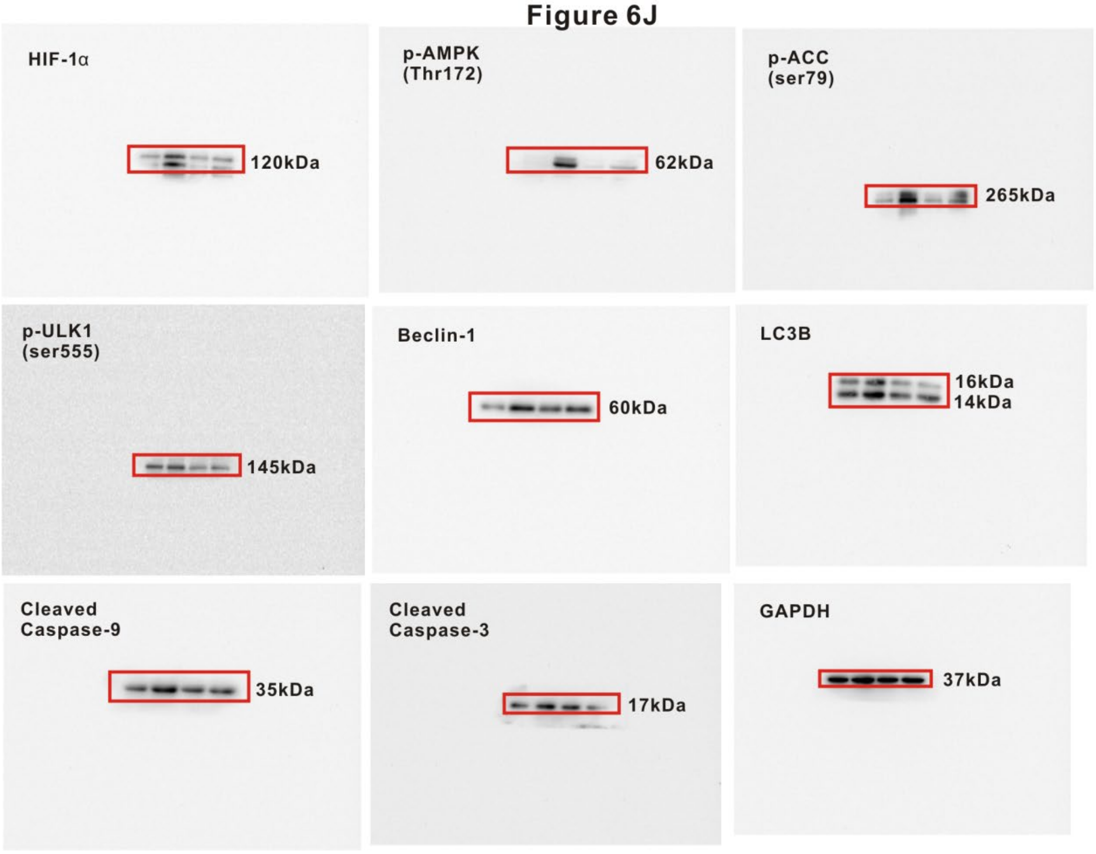

T24

Figure 7D

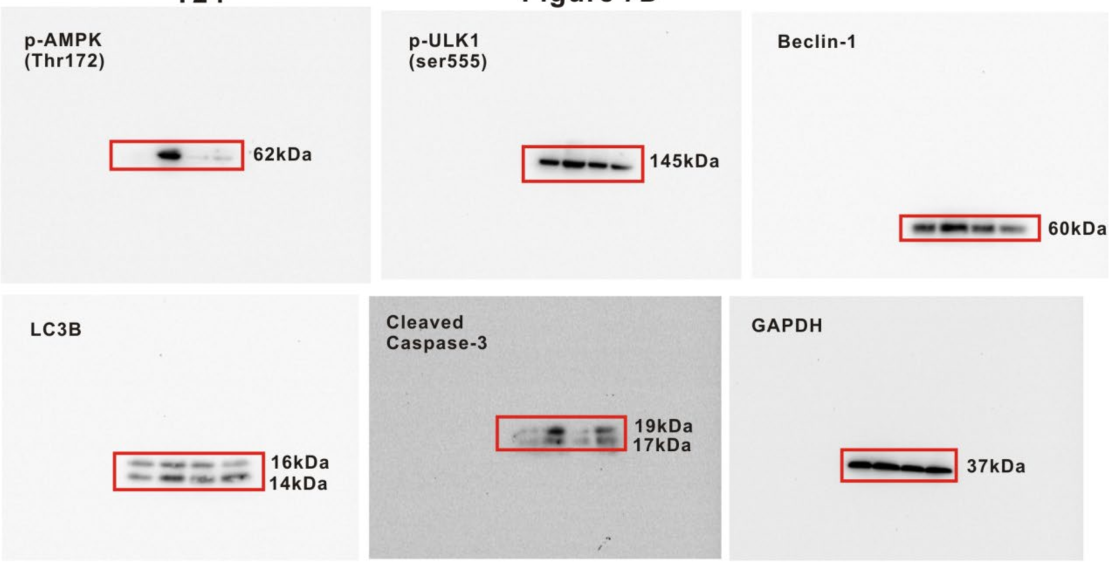

Figure 7E

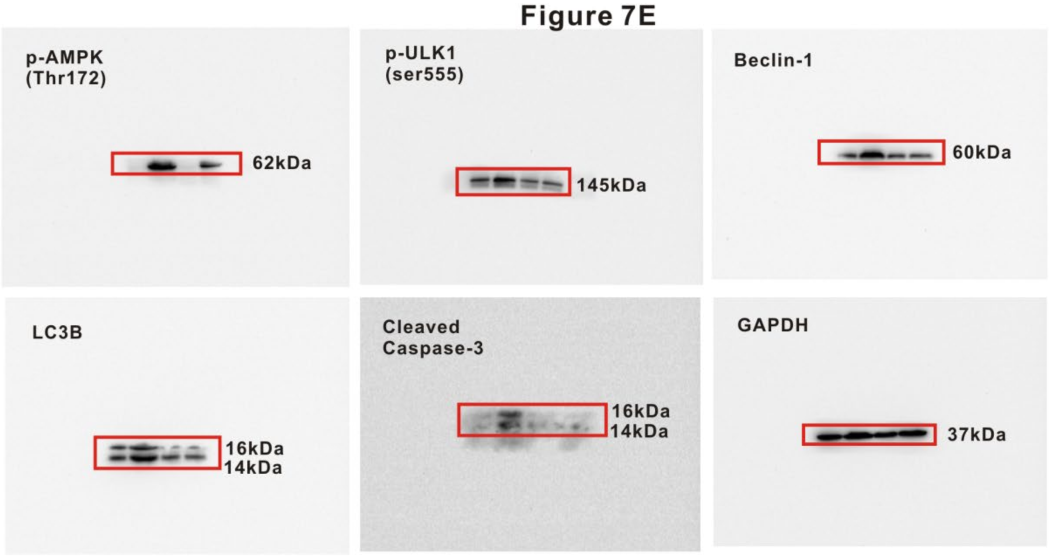

EJ

Figure 7F

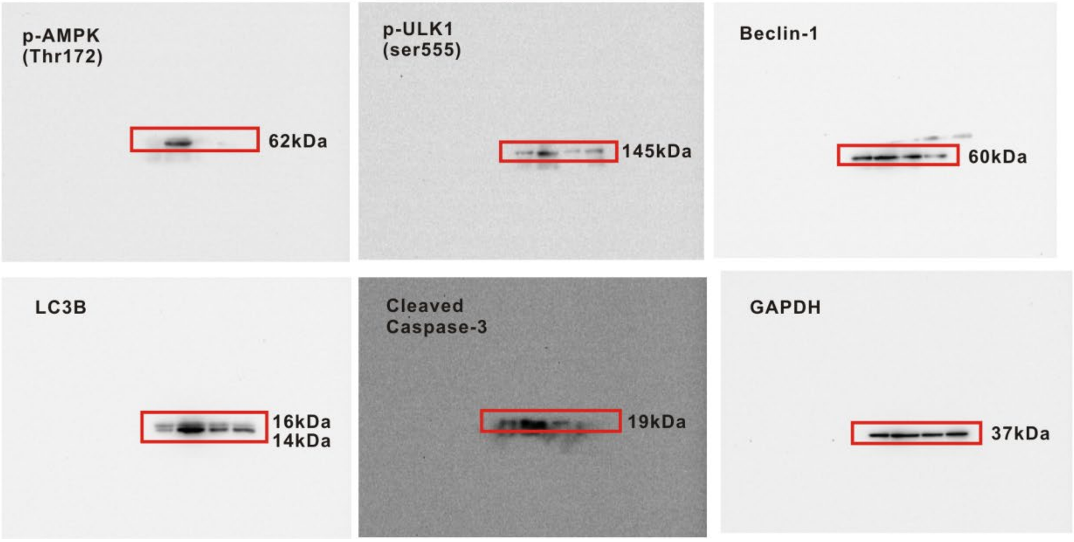

Figure 7G

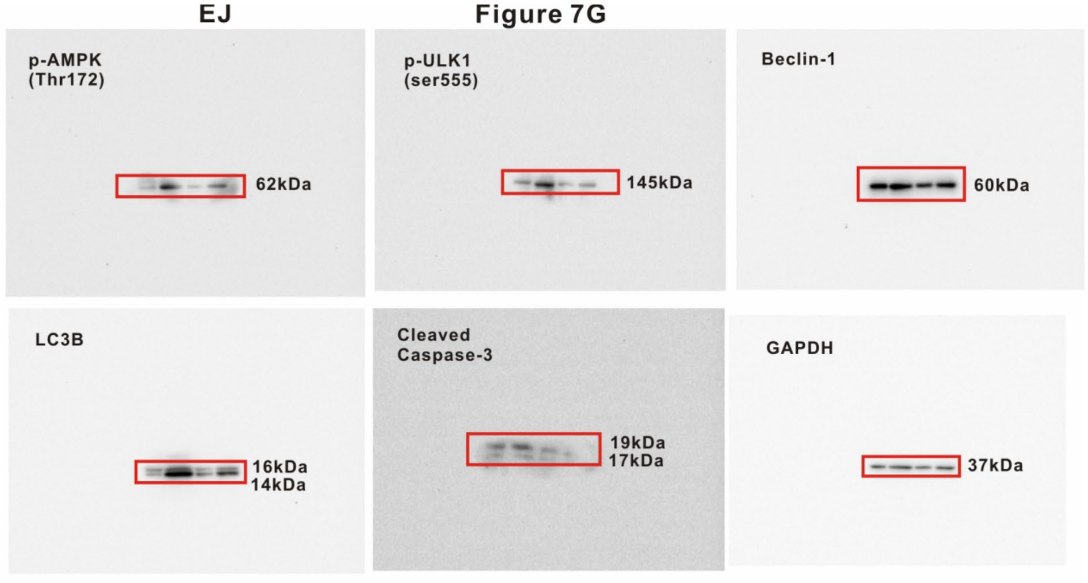

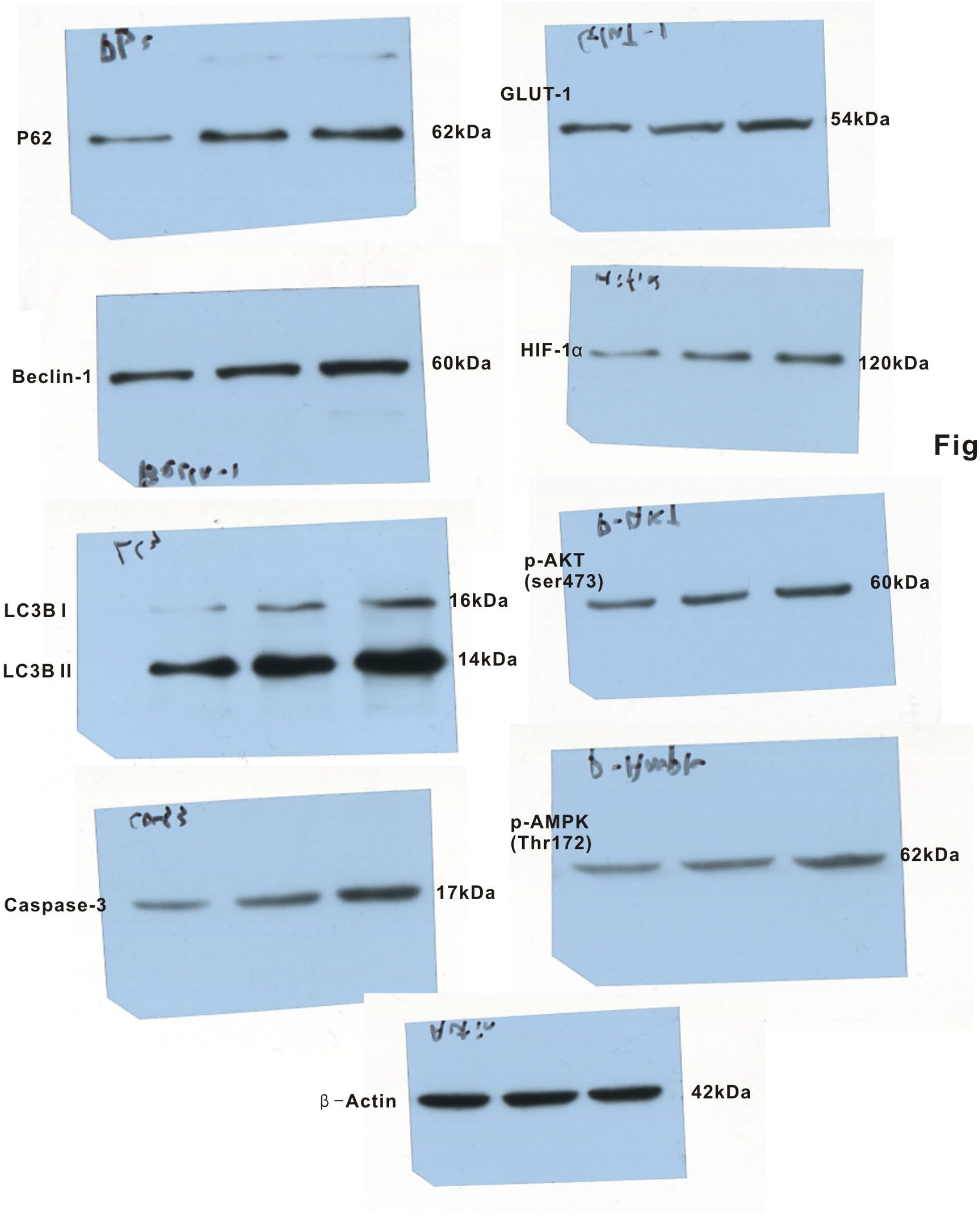

Figure 8F

Supplementary Figure 2A

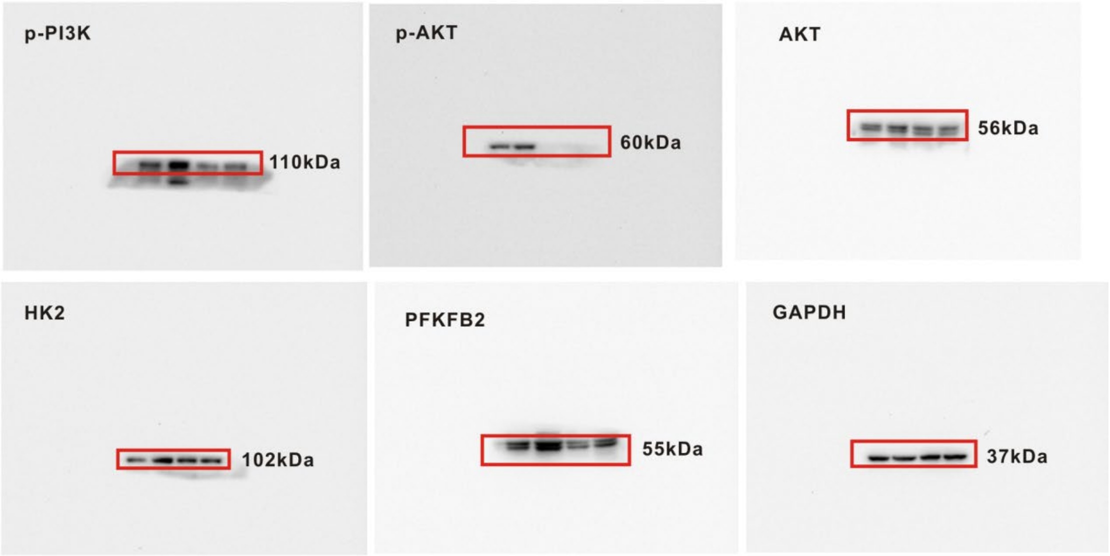

Supplementary Figure 3B

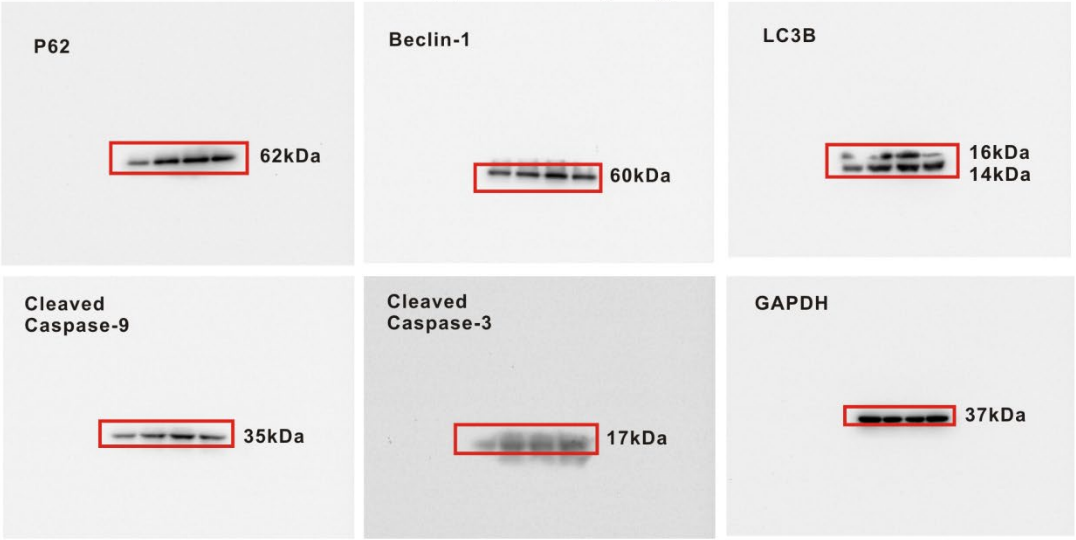

Supplementary Figure 3D

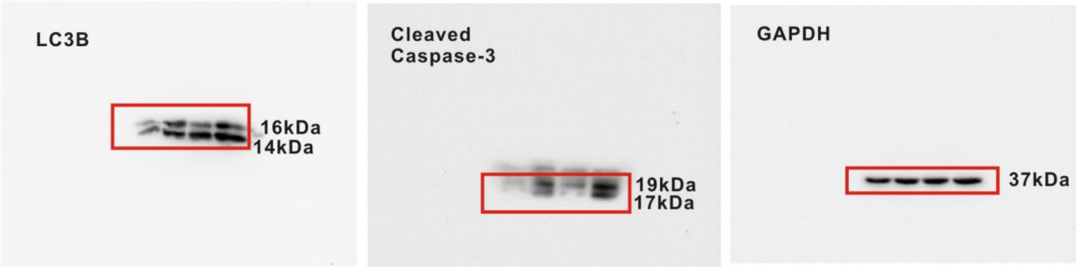

Supplementary Figure 4A

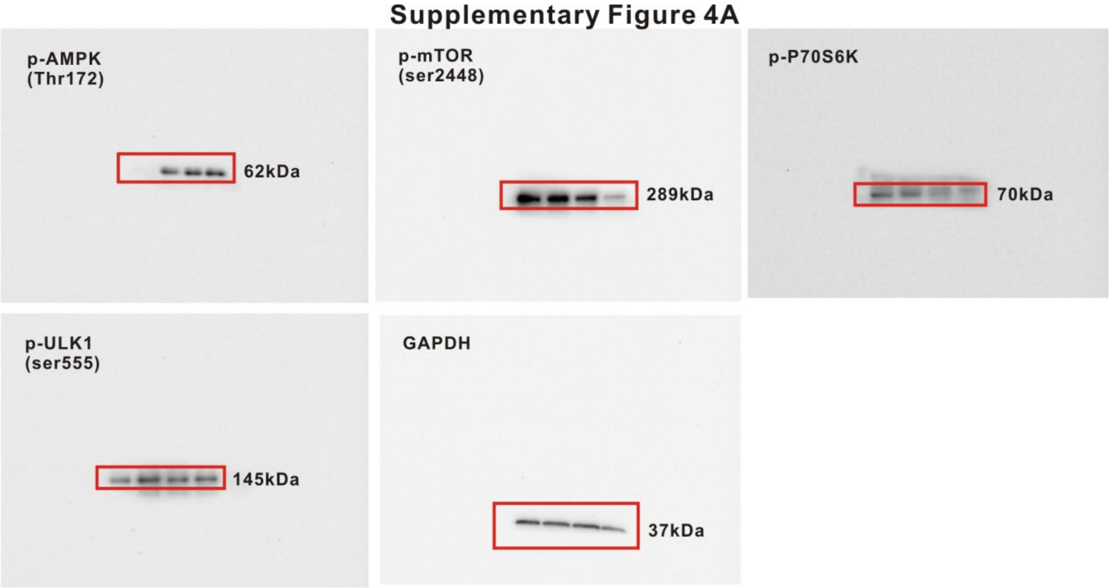

Supplementary Figure 4B

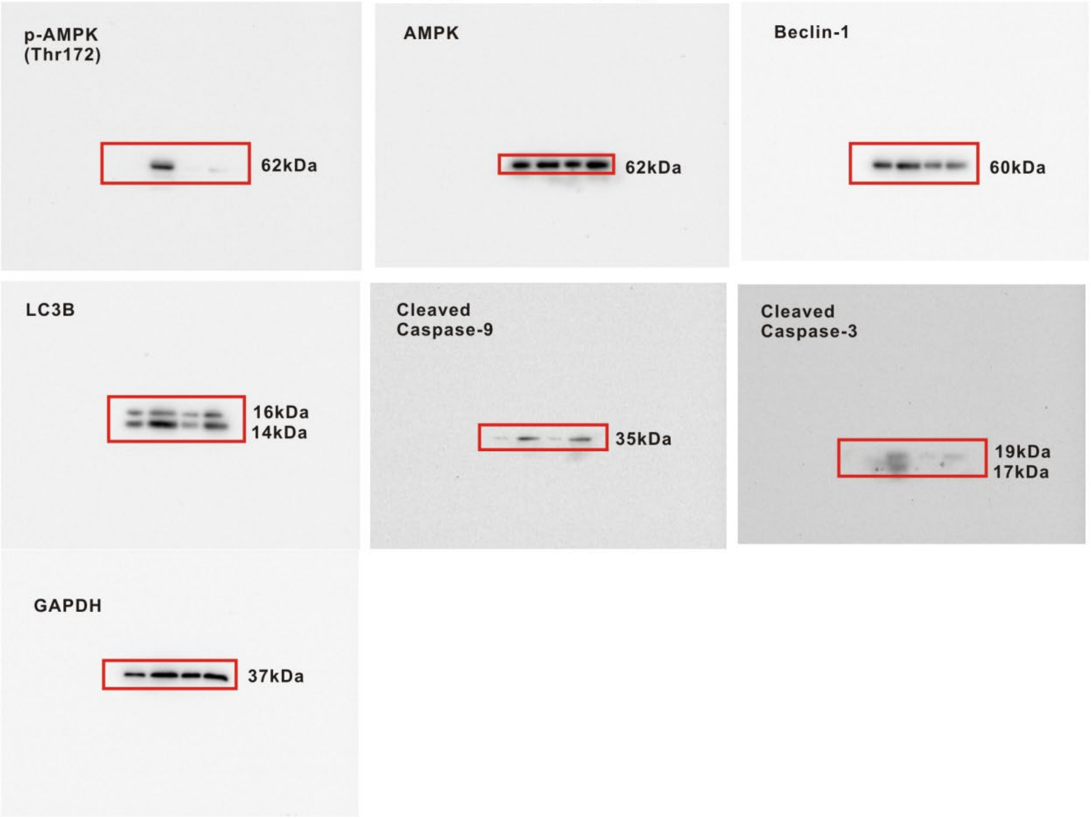

Supplementary Figure 5A

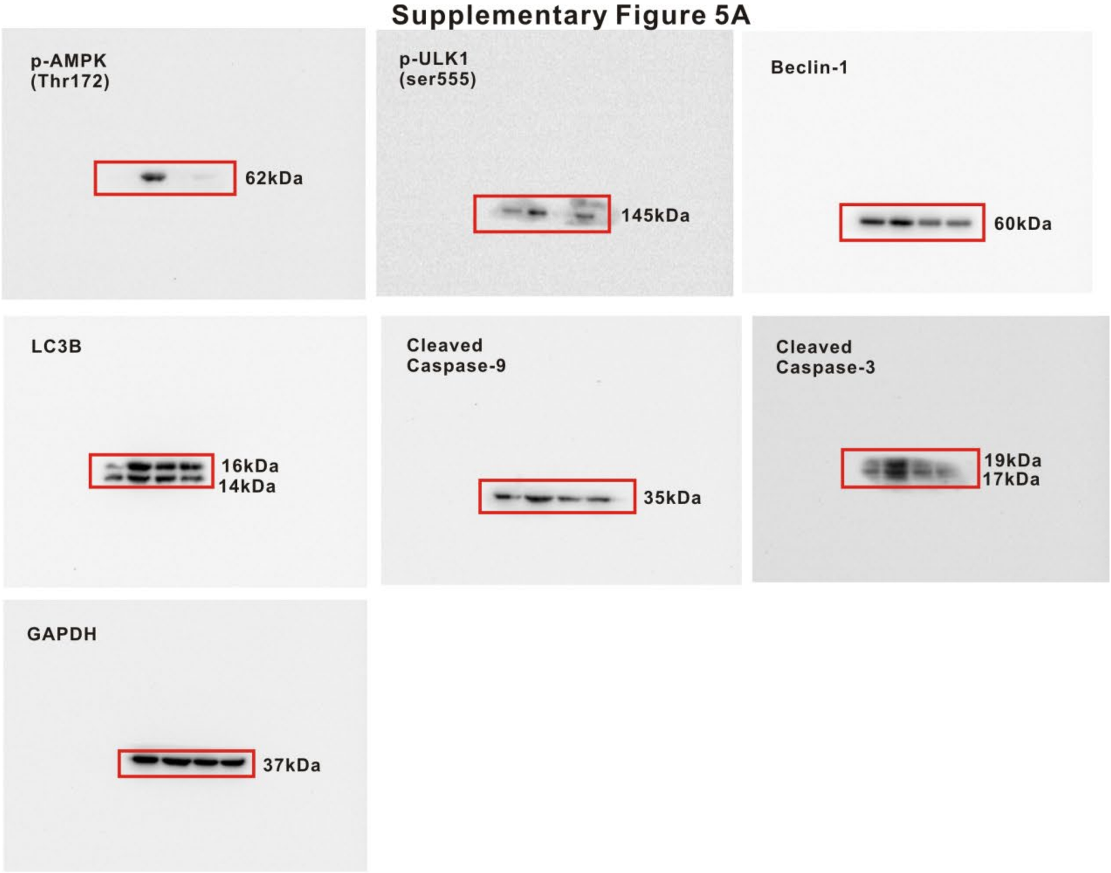

Supplementary Figure 6C

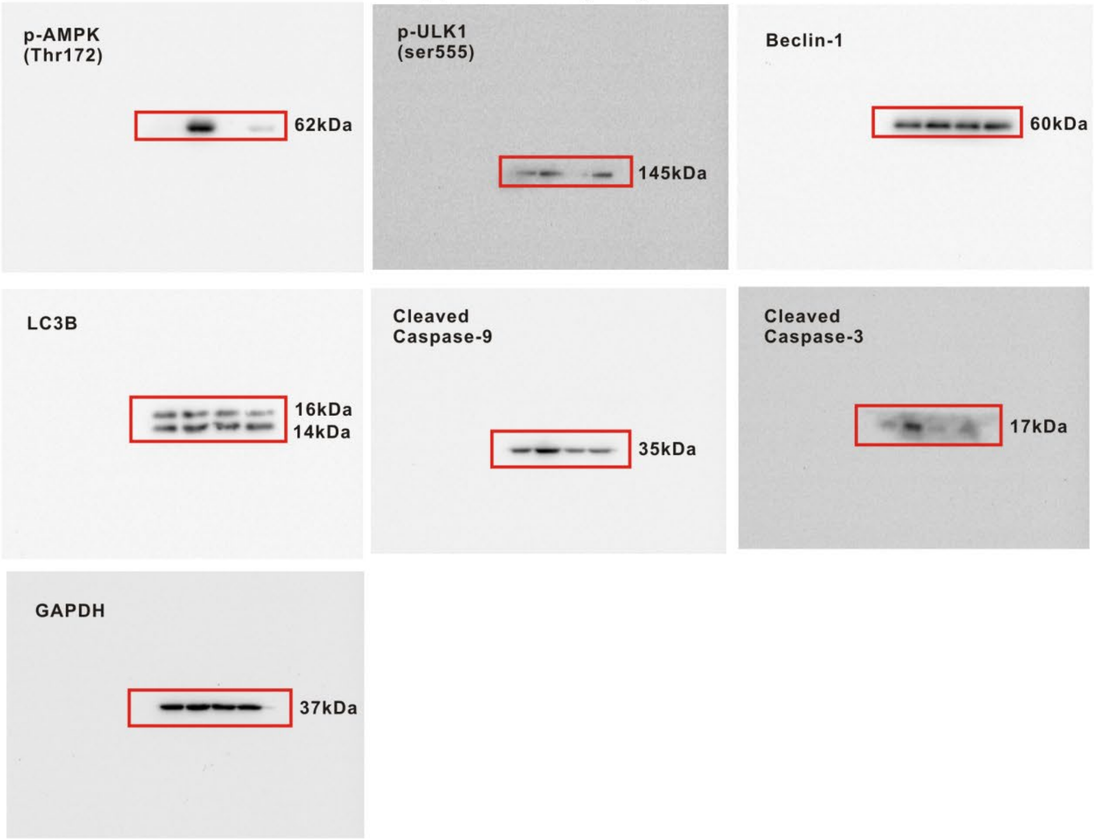

Supplementary Figure 7D

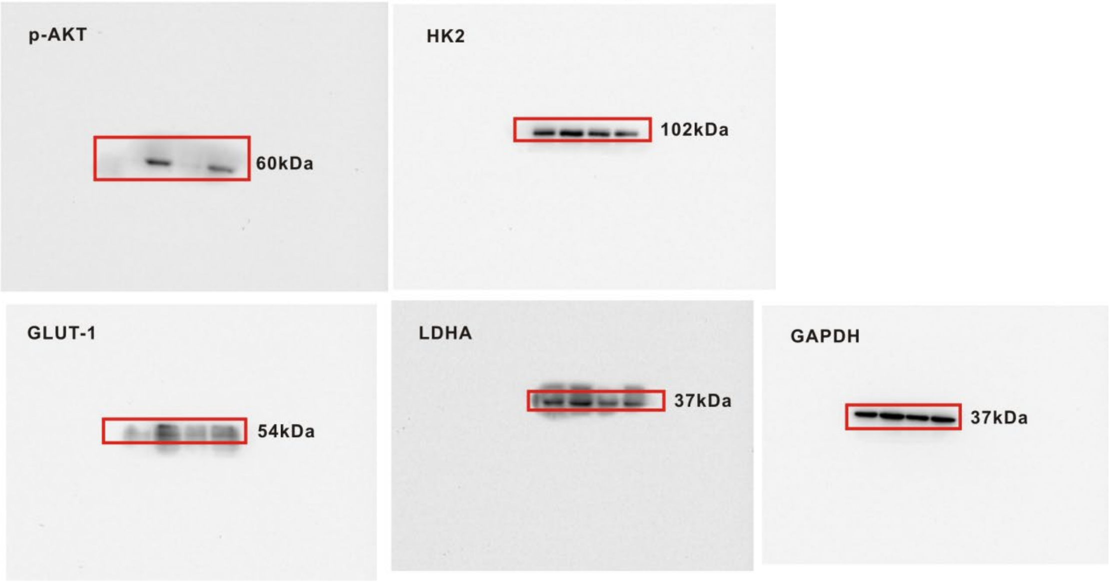

Supplementary Figure 7E

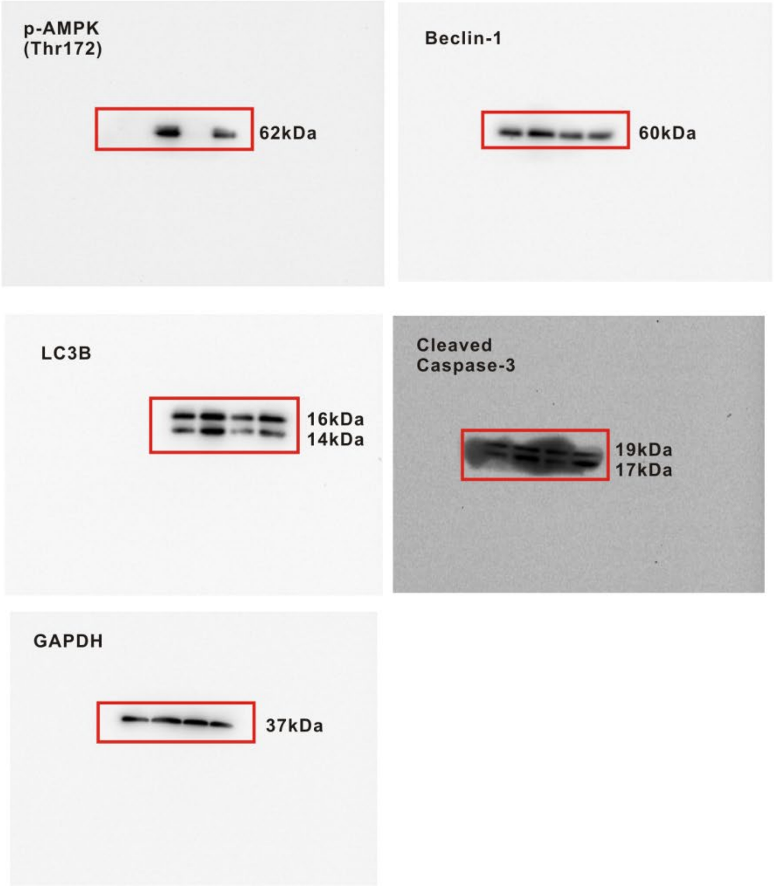

Supplement: Supplementary file 1 — Supplementary information. [file 41598_2020_64880_MOESM1_ESM.pdf]
